# Cooking Oil-Derived Aldehydes: Overlooked Components of the Western Diet with the Potential to Disrupt Fetal Brain Development

**DOI:** 10.3390/molecules31193530

**Published:** 2026-10-03

**Authors:** Catherine Shanahan, Martin Grootveld

**Affiliations:** Leicester School of Pharmacy, Faculty of Health and Life Sciences, De Montfort University, The Gateway, Leicester LE1 9BH, UK

**Keywords:** cytotoxic aldehydes, NMR analysis, lipid oxidation products, peroxidation, redox biology, oxidative stress, neurodevelopment, neuropathology, analytical chemistry, Dietary LOP Pathology

## Abstract

Dietitians routinely recommend refined high polyunsaturated fatty acid (PUFA) content culinary oils such as soybean, corn, canola, rapeseed and sunflower oils to pregnant women. Unfortunately, these PUFAs readily undergo peroxidation (especially at high frying or cooking temperatures), generating toxic lipid oxidation products, including reactive aldehydes and oxidized lipid mediators with established biological activities that may negatively impact fetal brain development. This hypothesis-driven perspective paper proposes a conceptual framework linking the maternal consumption of oils contaminated with lipid oxidation products to fetal neurodevelopmental vulnerability. We also identify key research priorities, including improved exposure assessments, the development of reliable aldehyde and other oxylipin biomarkers, and controlled dietary intervention studies. Such investigations will help clarify whether these commonly consumed culinary oils meaningfully contribute to the increasing rates of adverse neurodevelopmental outcomes.

## 1. Introduction: Pro-Oxidative Process Contaminants in Processed and Thermally Stressed Foods

The fetal brain is uniquely vulnerable to oxidative and electrophilic stress due to its high metabolic rate and high PUFA content. Multiple aspects of neurological development depend on tightly regulated redox signaling pathways, including the cytoskeletal dynamics that underpin structural developmental changes necessary for brain development. Oxidative and electrophilic stress, therefore, can produce lasting alterations in brain structure and function. Consequently, considerable research effort has focused on identifying environmental exposures capable of perturbing the developing nervous system by inducing oxidative and electrophilic stress. These include toxic metal ions [1], pesticides [2], tobacco smoke [3,4], air pollutants [5], pharmaceutical agents [6,7], and maternal inflammatory states [8,9], along with so-called Western diets, which are characterized by a high proportion of processed foods [10,11,12,13,14].

Despite current interest in the mechanisms linking processed foods to poor health outcomes, a conspicuous gap remains in understanding the biological effects of chemical transformations that occur during their industrial processing and preparation. While many investigators have discussed the possible role of intentionally added compounds such as preservatives [15], artificial colorings [16], and unintentional environmental contaminants such as pesticides [17,18], herbicides [19], and heavy metal ions [20], few have examined the chemical transformations that occur within the lipidic component of processed foods. Indeed, these may arise during the processing stage itself. This gap becomes especially important in the context of modern industrial food production, where manufacturers rely heavily on refined, polyunsaturated fatty acid (PUFA)-rich oils, and routinely expose them to high temperatures during processing and cooking episodes. These conditions generate high levels of lipid oxidation products (LOPs), including a range of toxic reactive aldehydes, and foods exposed to these conditions are prevalent in the Western diet [21]. Food chemists have long recognized that PUFAs are highly susceptible to oxidation, and have developed strategies—such as antioxidant stabilization—to limit the resulting degradation, primarily to preserve flavor, shelf life, and product quality [22]. However, the end goal of their work is largely to understand how to improve palatability and shelf life, rather than understanding the biological and health effects and potential toxicities of the LOPs themselves [23,24].

Intriguingly, there is one well-studied dietary pattern that excludes not just partially and fully hydrogenated fats, but also high-PUFA content cooking oils to a large extent—The Mediterranean Diet—which largely swaps these out for olive oil. Santacroce et al. [25], in their review of maternal factors impacting fetal inflammation and oxidative stress, report that maternal adherence to a Mediterranean Diet during the periconception period and pregnancy is associated with better infant social and autonomic functioning, including reduced depression, anxiety, and atypical behaviors, along with a lower risk of autism spectrum disorders. They further note findings from a randomized controlled trial of 90 pregnant women linking maternal Mediterranean Diet adherence to greater fetal total brain volume, corpus callosum size, and right frontal lobe volume, as well as improved autonomic stability scores in infants at 1–3 months of age. Although Santacroce et al. [25] highlight multiple maternal nutritional defects that may promote oxidative stress, they do not mention high-PUFA content oils, nor aldehydes, nor other LOPs as potential contributors [25,26]. This highlights just how rare it is for clinically oriented researchers to engage with the complex issue of lipid oxidation [27].

A separate body of research, led by investigators such as Martin Grootveld, Ameer Taha, and Gerhard Spiteller, among others, has demonstrated that LOPs, including reactive aldehydes, are absorbed into the systemic circulation and can exert a myriad of direct and indirect toxicological effects *in vivo* [28,29,30,31,32,33,34,35,36]. Such investigations have collectively shown that different types of LOPs can exert directly toxic effects by covalently reacting with proteins, DNA, and other cellular biomolecules. In addition, various LOPs can promote cellular oxidative stress, which pathologists have identified as playing a role in most disease states by disrupting protein folding, membrane biology, enzyme function, cellular signaling, energy production, and other fundamental cell processes. Notably, disrupting these cellular activities has been shown to disturb cell development and migration, and it is plausible that these may lead to a variety of neurologic anomalies seen in perinatal brain development disorders, as reviewed in detail below.

One thing that has confounded the field is the fact that many of the same aldehydes present in dietary oils can also form endogenously during the *in vivo* peroxidation of polyunsaturated fatty acids (PUFAs), with mitochondrial oxygen radical generation representing a principal source [34,35,36]. The body defends against this endogenous aldehyde burden with a complement of antioxidants, including enzymes, vitamins, and glutathione, amongst others. Thus, heated culinary oils may exert an additional layer of toxicity by stressing the body’s antioxidant defenses, leading to a state of chronically heightened exposure to mitochondrial (and other) sources of reactive oxygen species (ROS). A representative list of culinary oils available in the “Western world” indicating percentages of peroxidizable MUFAs and more susceptible PUFAs, respectively, is: avocado oil 74% and 14% (*w*/*w*); butter 30% and 4% (*w*/*w*); canola oil 62% and 32% (*w*/*w*); coconut oil 6% and 2% (*w*/*w*); corn oil 25% and 62%; olive oil 73% and 11%; palm oil 38% and 10%; safflower oil 14% and 75% (*w*/*w*); soybean oil 23% and 58% (*w*/*w*); sunflower oil 20% and 69% (*w*/*w*); and tallow 50% and 4% (*w*/*w*). The remainder is predominantly composed of saturated fatty acids, as their triacylglycerol equivalents. Hence, those with the highest PUFA contents (soybean, corn, sunflower and safflower oils) are potentially the most hazardous to use for frying or cooking purposes.

Despite a growing body of mechanistic and analytical work, the literature encompassing the role of LOPs in promoting cell dysfunction relevant to embryologic life remains only loosely connected to mainstream nutrition science and epidemiologic research, and has not yet been systematically incorporated into prevailing eating pattern frameworks. Indeed, high PUFA content culinary (cooking) oils are generally considered beneficial, and this perception has perhaps contributed to the relative dearth of focus on cooking oil-derived LOPs. At the same time, unhealthy dietary patterns, particularly those high in processed foods, snack foods, and fried foods, and often characterized as “Western-style” dietary items, have been associated with a wide range of adverse health outcomes, including obesity, type 2 diabetes, cerebrovascular and coronary vascular diseases, cancer, mental illness, and neurological degeneration [37,38], amongst others. Maternal diets fitting this latter eating pattern have been linked to disrupted brain development in human and animal studies [1,2,3,4,5,6,7,8,9,10,11,12,13,39,40,41,42]. Notably, the Western diet has been causally linked to oxidative stress, which is believed to be a key factor driving such poor health outcomes. However, the literature available on the Western diet reflects confusion about the nature of the most prevalent types of fatty acids present. Some authors focus on saturated fat, such as Clemente-Suárez (2023) [21], who define a Western diet eating pattern as “characterized by high intakes of processed and refined foods, red and processed meats, added sugars, and saturated and *trans*-fats”, while others, such as Blasbalg (2011) [43], recognize that the dominant fatty acids in the food supply have shifted, so that PUFAs compose a considerably larger portion than was the case a few short generations ago, with linoleic acid (LA) being the single most prevalent one. This persistent misunderstanding about the nature of the lipid component of a Western, processed-food-rich diet is particularly problematic given the lifelong impact of neurodevelopmental disorders and their currently growing burden being placed on society to assist with the proper care of children and adults with special needs.

Yet another source of confusion in the clinical nutrition literature regarding the potential harms of high PUFA content oils comes from focusing on linoleic acid’s (LA’s) metabolic effects as an omega-6 fatty acid rather than focusing on lipid chemistry. The nutrition literature has engaged with the fact that soybean oil, and other seed oils, are high in LA, and that rising consumption of these oils has altered the fatty acid composition of human body fat so that it now contains much more LA than in decades past. However, the mechanistic concern most discussed is the fact that LA is a precursor to the omega-6 series of inflammatory eicosanoids, and its consumption may adversely promote chronic inflammation. However, this omega-6 hypothesis has been somewhat nullified. Indeed, a systematic review of 15 randomized controlled trials found little evidence that increasing dietary LA raises inflammatory marker concentrations in healthy adults [44]. In our opinion, the eicosanoid framing also misses what we consider a far more consequential mechanism, namely that PUFAs (including both the omega-6 and omega-3 fatty acid families) serve as direct chemical precursors to LOPs, which include highly toxic aldehydes. These LOPs form in high PUFA content culinary oils and are well characterized, as discussed in Section 2. Notably, the same oxidative chemistry also applies in biological systems. This would particularly affect PUFAs present in membrane phospholipids, lipoproteins, and triacylglycerols. However, the degree to which dietary LOPs can promote *de novo*, *in vivo* LOP formation and oxidative stress is critically under-examined. In view of its high PUFA content and high metabolic activity, the brain is particularly vulnerable to LOP exposure and oxidative stress.

Therefore, this hypothesis-driven perspective paper also invites the perinatal nutrition research community to recognize and more seriously consider the impact of dietary LOPs on endogenous PUFA oxidation because the endogenous formation of toxic LOPs may negatively impact cellular enzyme activity, cytoskeletal dynamics, and redox balance. These potential consequences of *in vivo* lipid peroxidation become especially consequential in light of the roughly 2.4-fold increase in adipose tissue LA content documented between 1959 and 2008 [45]. The historical changes may be far greater, as illustrated by a 1975 study in the Netherlands performed before the import of high PUFA content oils [46]. The authors evaluated adipose PUFAs of formula-fed infants given a maize-oil-based feed. They found adipose LA rose from 1 to 3% at birth to 32–37% by four months of age, compared with a rise to no more than 3% in infants fed a cow’s-milk-based formula over the same period. Whether accumulating gradually across a population over decades, or acutely within an individual over months, this expansion of the endogenous PUFA substrate pool substantially increases the material available for peroxidation throughout the body. This necessarily increases the risk of diseases associated with oxidative stress, unless the body is able to adapt to the chronic radical and oxidative exposures, which is less likely on a typical “Western” diet.

This paper therefore puts forth the postulate that cooking oil-derived LOPs, particularly aldehydes, that are prevalent in processed, snack and fried foods, collectively represent powerful oxidative stress-inducing components that warrant consideration as potential drivers of diet-induced disease. Moreover, their presence in a maternal diet may stress the developing fetal brain. Beyond their detection in snack and fried foods, LOP-forming reactions also take place in these oils when used to prepare fried or cooked foods in the home, or in restaurants—fast-food or high-end. Oil frying operations often take place between temperatures of *ca.* 160–230 °C (320–450 °F), where UFA degradation takes place quite rapidly [47,48]. Hence, the oil temperature could exceed the above recommended 180 °C whilst remaining below the advertised “smoke point” of many culinary oils. Furthermore, many foods contain metal ions that may act as catalysts for lipid oxidation of PUFAs, and could accelerate LOP formation so that even relatively short cooking times could lead to significant formation [49,50]. Reactive aldehydes represent a particularly informative sub-set of toxins, because they are systemically bioavailable and highly electrophilic, enabling them to form covalent adducts with protein, amino acid and nucleic acid nucleophiles, for example. These properties render aldehydes one of the most biologically active and experimentally traceable mediators of lipid peroxidation, and thus a useful focal point for examining the potential developmental consequences of the adverse health effects of dietary LOPs. To support the potential link between cooking oil-derived LOPs and adverse neurological perinatal outcomes, we begin by highlighting the prevalence of these overlooked dietary components. We then synthesize evidence from toxicology, lipid chemistry, developmental neurobiology, and maternal nutrition. We further highlight specific neurodevelopmental processes that aldehydes are known to disrupt, outline the implications of these mechanisms for cortical development, and propose a targeted research agenda to determine whether or not these electrophiles constitute a modifiable environmental contributor to adverse neurodevelopmental outcomes.

## 2. The Ubiquity of Oil-Derived Reactive Aldehydes in Modern Maternal Diets

Modern dietary patterns expose women of reproductive age to cooking (predominantly seed) oil-derived LOPs far more frequently than in previous generations [43]. This shift is driven in large part by the widespread adoption of refined high-PUFA content cooking oils beginning in the late 1950s. In the USA particularly, soy oil consumption in particular took off, having increased from less than 2 kg/person/year prior to 1960 to nearly 12 kg/person/year in 1999 [51]. Consumption of liquid (non-hydrogenated) soy and other high PUFA content cooking oils has undoubtedly increased further since 1999 because of the relatively recent *trans*-fat ban; however, there has been little published on the matter recently in view of the cessation of US governmental tracking programs. Around the world, these oils now supply a substantial proportion of total dietary fat. The most prevalent PUFA in cooking oils is the omega-6 fatty acid LA, the concentration of which ranges from a low of ~20% (*w*/*w*) in canola to a high of ~74% (*w*/*w*) in safflower oils. The second most prevalent PUFA is the omega-3 fatty acid alpha-linolenic (ALA), with concentration ranging from 0.1 to 0.2% in sunflower, to approximately 2% (*w*/*w*) in cottonseed, rice bran, safflower and corn oils, and up to ~9% (*w*/*w*) in canola and rapeseed oils [52].

**Figure 1:**
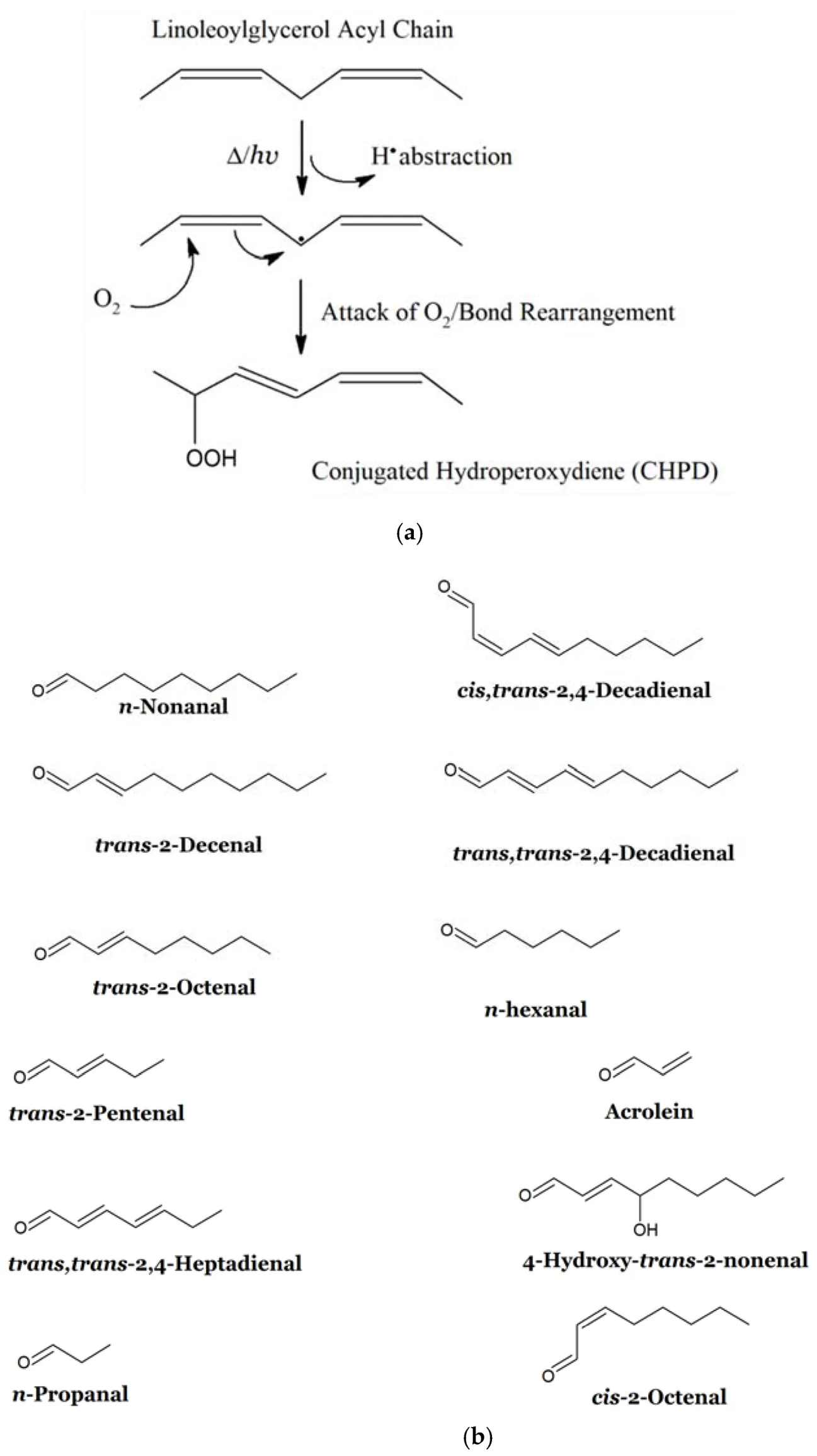
(**a**) Reaction process for the peroxidation of a LA substrate molecule present in a culinary oil linoleoylglycerol species (H● represents a hydrogen atom). The conjugated hydroperoxydiene (CHPD) aldehydic precursor shown is one of the (*Z*,*E*)-CHPD types, although (*E*,*E*)-CHPD isomers are also produced. (**b**) Molecular structures of aldehydic LOPs derived from the fragmentation of lipid hydroperoxides, both hydroperoxymonoenes (HPMs) (from oleoylglycerols) and CHPDs (from linoleoylglycerols). *n*-Nonanal and (*E*)-2-decenal arise from the degradation of oleoylglycerol-derived HPMs, whilst *n*-hexanal, (*E*)-2-octenal and (*E*,*E*)-deca-2,4-dienal arise from the fragmentation of linoleoylglycerol-derived CHPDs. Propanal, acrolein, (*E*)-2-pentenal and (*E*,*E*)-hepta-2,4-adienal are derived from linolenoylglycerol CHPD deterioration. One route for the generation of (*Z*,*E*)-deca-2,4-dienal is the thermal isomerization of its (*E*,*E*)-isomer. This diagram was originally published in Ref. [29] and is reproduced here in full.

The vast majority of high PUFA content oils in the food supply chain have undergone extensive refining that removes choline, a brain-critical nutrient, and also removes and/or oxidizes alpha-tocopherols, another brain-critical dietary agent. Further key agents lost or diminished in concentration are many of the natural antioxidants that normally serve to slow or perturb oxidative degradation of peroxidizable, PUFA-rich oils [53,54]. As a result of the refining process, these oils are rendered relatively non-nutritive, with an enhanced susceptibility to oxidation. However, the most pertinent characteristic of them relates to their high PUFA content, which is the most important variable to consider when it comes to oxidative degradation. In view of this combination of factors particular to these oils, they are indeed extremely prone to rapid oxidative breakdown upon exposure to light, air, and heat, and their continuous exposure to high temperatures during the production of a range of deep-fried restaurant and prepared foods is especially damaging [55,56]. The oxidative breakdown of PUFAs in these oils transforms them into a multitude of new, “foreign” molecules, most of which have toxic properties [57]. Amongst them, it is generally agreed that the *alpha*,*beta* (α,β)-unsaturated aldehydes offer the highest level of reactivity in biological systems, and thus are the most directly toxic [58]. Figure 1 provides schemes for generation of the major *alpha*,*beta*-unsaturated aldehydes, along with saturated aldehydes, and their conjugated hydroperoxydiene precursors, which are derived from specific PUFAs prevalent in high-PUFA content culinary oils. Please note that the isomeric specifications for these *cis*- and *trans*-LOP isomers are denoted by the bracketed italicized letters (*Z*)- and (*E*)-, respectively (Table 1).

Typical molar percentage levels of the wide range of aldehydes generated are 30.5 ± 2.4% for (*E*)-2-alkenals; 27 ± 2.8% for (*E*,*E*)-alka-2,4,-dienals; 17 ± 1.65% for *n*-alkanals; 8.5 ± 0.4% for (*Z*, *E*)-alka-2,4-dienals; 7.5 ± 1.0% for 4,5-epoxy-(*E*)-2-alkenals; 5.5 ± 0.6% for combined analytical admixtures of 4-hydroxy- and 4-hydroperoxy-(*E*)-2-alkenals; 2 ± 0.3% for 4-oxo-*n*-alkanals; and 1 ± 0.4% and 1 ± 0.2% only for both (*Z*)-2-alkenals and low-molecular-mass *n*-alkanals, respectively. These data are from a study involving the thermal stressing of a commercial soybean oil product at 180 °C for a 90 min duration. It is also of much importance for readers to take into consideration that a series of the aldehydic LOPs generated are quite or very volatile, with many of them having boiling points (b.pts) lower or much lower than that of the standard frying temperature. Hence, the direct inhalation of these species during high-temperature frying episodes by humans in the kitchen at home, at restaurants or by workers at other sources, etc., can also play important roles regarding the route of their toxicological actions. A list of these b.pts is shown in Table S1, and it can be seen that many of them have values < 180 °C.

**Figure 2:**
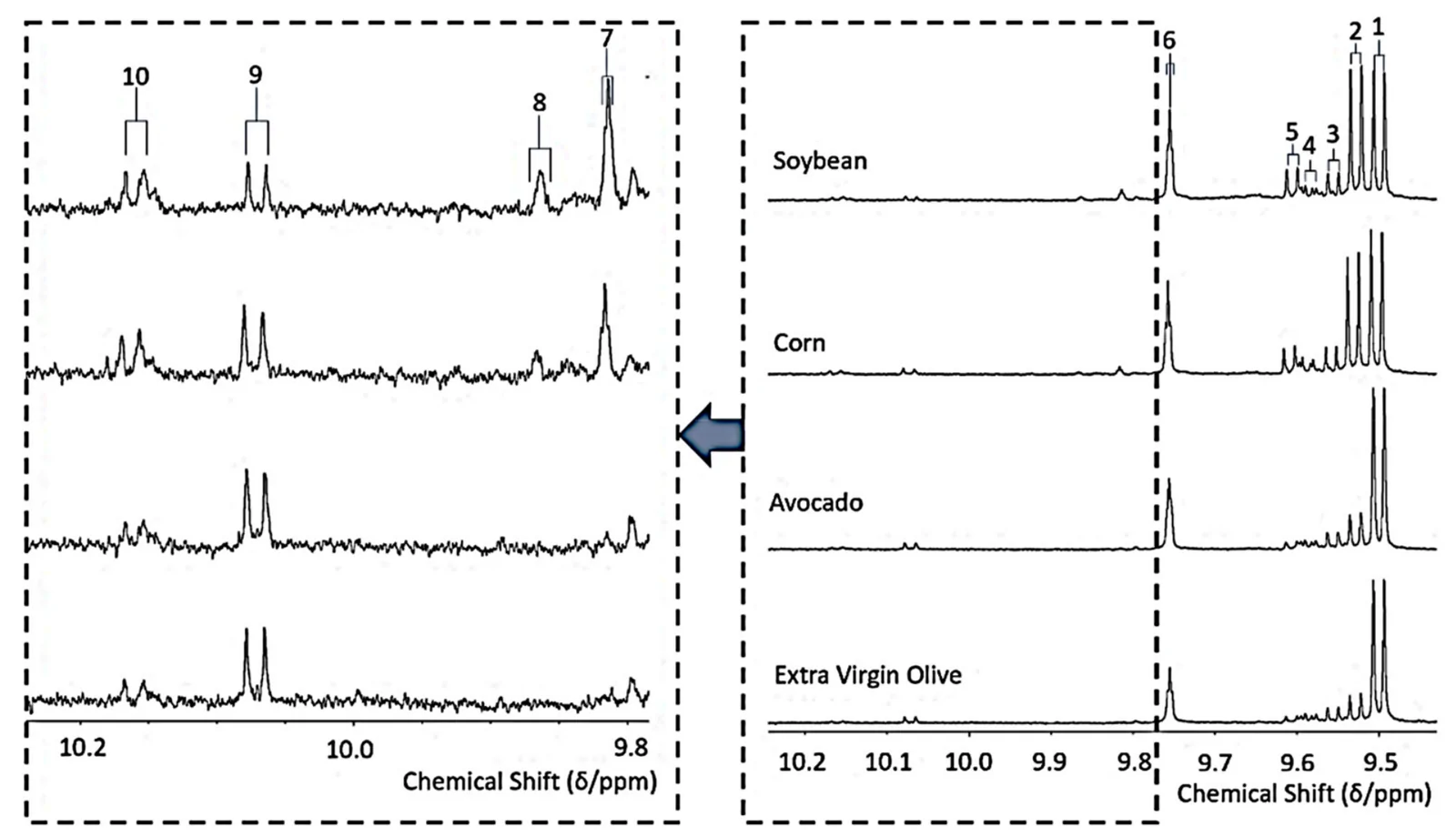
Aldehydic-CHO proton (9.44–10.22 ppm) regions of 600 MHz ^1^H NMR spectra of typical culinary oils exposed to thermal stressing episodes at 180 °C for a period of 90 min according to our laboratory-simulated shallow frying episodes (LSSFEs); the 9.79–10.22 ppm expanded regions of these spectra are shown on the left-hand side. Numerical abbreviations: as in Figure 2, with 8 representing low-molecular-mass *n*-alkanals (triplet) such as propanal, which predominantly arises from the peroxidation of linolenoylglycerol substrates available in soybean oil. Sample preparation and heating conditions: as in Figure 2. This diagram was originally published in Ref. [47] and is reproduced here in full.

**Figure 3:**
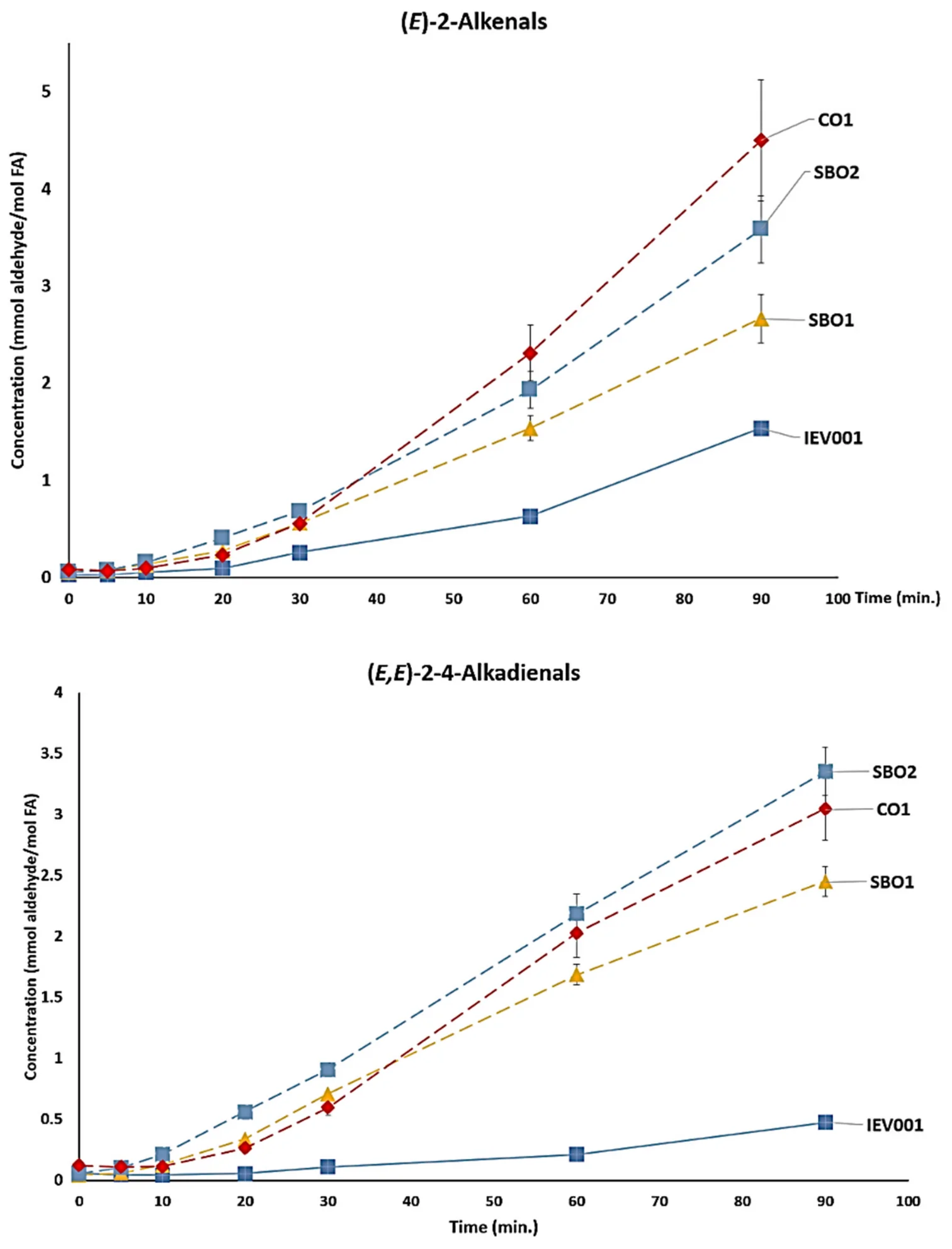

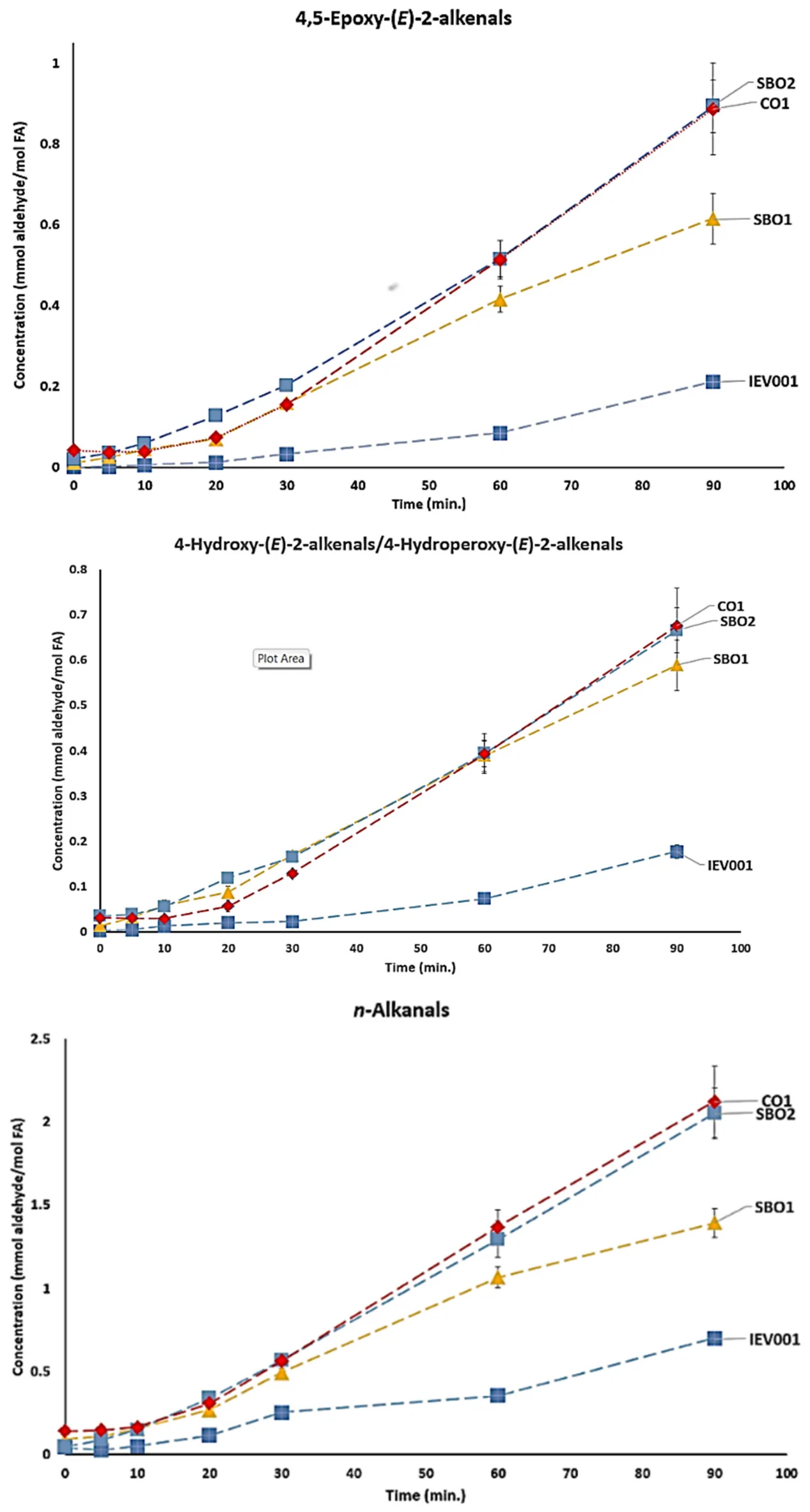

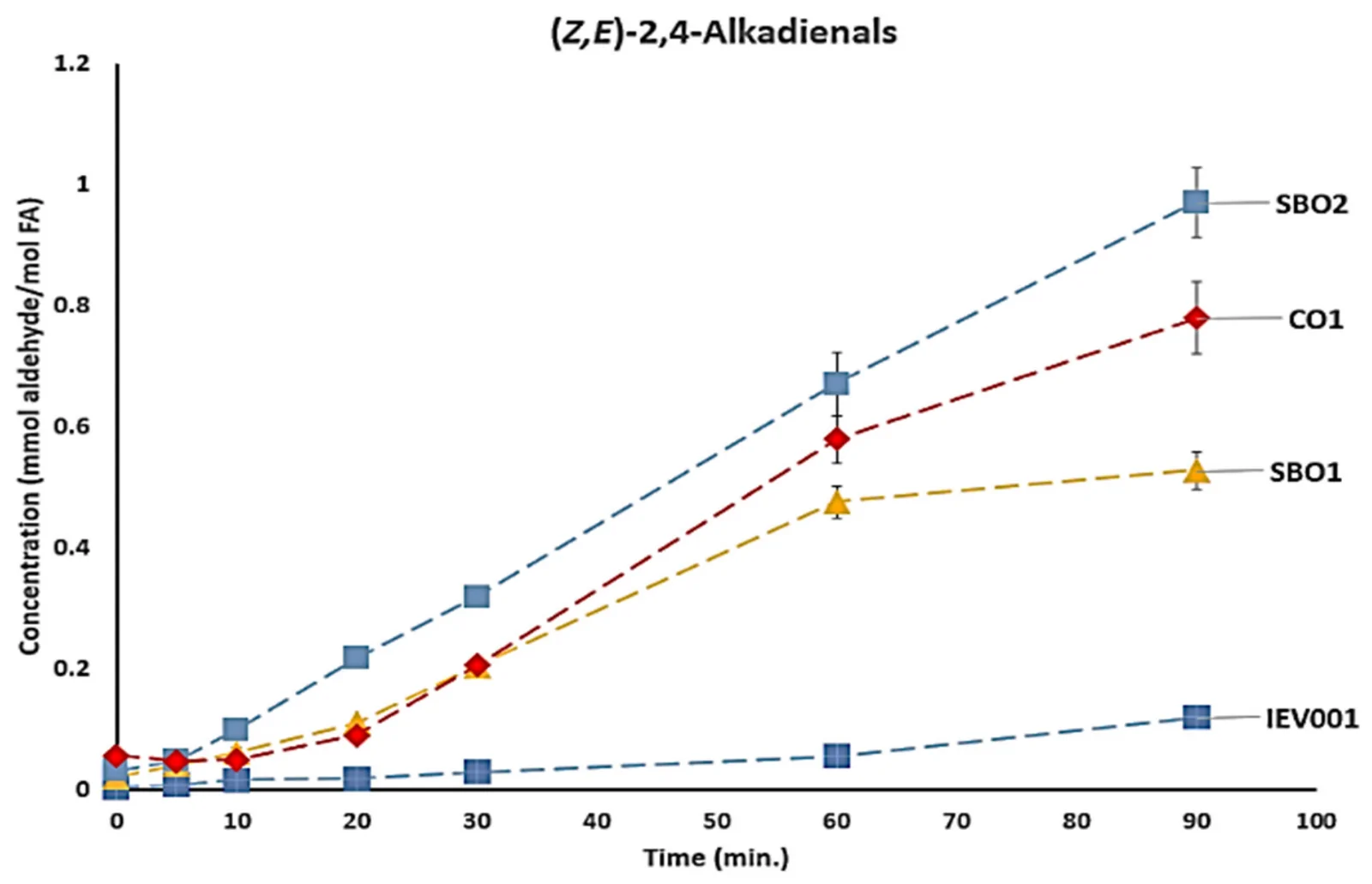
Plots of mean ± SEM concentrations of six replicate determinations of aldehydes (mmol/mol FA) versus heating times at 180 °C for a series of commercially available culinary oil samples thermally stressed according to our LSSFE protocol. These aldehydes are either unsaturated, *trans*-2-alkenals, *trans*,*trans*- and *cis*,*trans*-alka-2,4-dienals, and 4,5-epoxy-*trans*-2-alkenals, and an admixture of 4-hydroxy- and 4-hydroperoxy-*trans*-2-alkenals, whereas the saturated aldehydes included long-chain *n*-alkanals; with the exception of 4-hydroxy- and 4-hydroperoxy-*trans*-2-alkenals, all are ^1^H NMR-distinguishable. Oil heating and sample preparation conditions were those described in Figure 2. The label-specified total SFA, MUFA and PUFA contents (*w*/*w*) of the unheated (control) oils were: Italian extra-virgin olive oil (IEVOO1) 17.0, 76.0 and 7.0%; corn oil (CO1) 12.9, 27.6 and 59.5%; soybean oil 1 (SBO1) 16.8, 26.9 and 56.3%; soybean oil 2 (SBO2) 17.7, 24.1 and 58.2%, respectively. Of their total PUFA contents, products SBO1 and SBO2 contained 7.2 and 7.5% (*w*/*w*) linolenate, respectively, and therefore their linoleate contents were 49.1 and 50.7% (*w*/*w*) by deduction, respectively. SBO1 was described as an organic expeller-pressed vegetable oil, whilst SBO2 was a standard refined soybean vegetable oil. The sigmoidal relationships between aldehyde class concentrations and heating time-points are consistent with their autocatalytic, self-perpetuating generation. This diagram has been modified from that originally published in Ref. [47]. Samples were prepared for ^1^H NMR analysis as described in Figure S1. This Figure shows the expanded aldehydic-CHO proton region of the 600 MHz ^1^H NMR spectra of a typical canola oil product exposed to thermal stressing episodes according to our LSSFEs for increasing periods of time (0–90 min). Moreover, Figure S2 again shows this spectral region, but for an extract of a UK-available packeted potato crisp snack product, demonstrating the detection of four classes of aldehyde LOPs therein. Abbreviation: FA, fatty acid.

Readers should also note that the aldehydic species generated may seem unusual to them, but this is largely because much of the scientific literature available erroneously focuses on only 4-hydroxynonenal (HNE) and malondialdehyde (MDA), which only have a relatively low yield from the lipid peroxidation process, unlike selected *E*-2-alkenals and (*E*,*E*)-alka-2,4-dienals, which are generated at higher or much higher levels (Figure 2 and Figure 3). It should also be noted that some ^1^H NMR-detectable aldehydes have Thresholds of Toxicological Concern (TTC) values that are significantly lower than those of even HNE, which is 1.5 µg/kg bw (Table 1). For example, the oxygenated aldehydes 4,5-epoxy-(*E*)-2-octenal and 4-hydroperoxy-(*E*)-2-octenal, the classes of which are readily generated from the peroxidation of PUFAs, and which are produced at total levels that are similar to those of total 4-hydroxy-(*E*)-2-alkenals (Figure 2 and Figure 3), have TTC values as low as 0.0025 µg/kg bw. Moreover, the *E*,*E*- and *Z*,*E*-isomers of decadienal are also classified with the same TTC value as both HNE and 4-hydroxy-(*E*)-2-hexenal (HHE). TTC values represent a level of human exposure to a chemical agent below which there is no appreciable risk to health, and these are set by the European Food Safety Authority [59].

### 2.1. Aldehydes from Omega-6 Linoleic Acid (LA)/Linoleoylglycerols

It is very important to note that the percentage yields of volatile aldehydic LOPs derived from LA/linoleoylglycerol substrates are not easily predictable, since the peroxidation process can yield very large numbers of these secondary products at variable rates of generation, and the identities and levels of those produced are critically dependent on O_2_ availability, temperature and reaction period [63,64,65].

Indeed, degradation of the primary hydroperoxides of LA/linoleoylglycerols (9- and 13-hydroperoxides) gives rise to a characteristic series of aldehydic LOPs, the relative proportions of these depending on the specific marker [65,66]. The most important and prevalent aldehydes generated from the thermal stressing of linoleoylglycerol-rich culinary oils are (*E*)-2-octenal, (*E*)-2-heptenal, (*E*)-2-nonenal, (*E*,*E*)-alka-2,4-decadienal and *n*-hexanal. n-Hexanal is the most abundant saturated aldehyde formed from autoxidised LA species, and (*E*,*E*)-alka-2,4-decadienal is often found at similar concentrations. Further aldehydic LOPs produced include 4-hydroxy-(*E*)-2-nonenal, which, although quite toxicologically significant, is generated only in relatively small amounts. We highlight this because, since (*E*)-2-alkenals and (*E*,*E*)-alka-2,4-dienals are the quantitatively dominant species found in thermallystressed, PUFA-rich culinary oils, it appears that many researchers involved in this research field have unfortunately been misled by focusing on HNE and MDA only. Hence, neurodevelopmental evidence for the above dietarily rich species is required. LA lipid derivatives also serve as sources of another highly reactive aldehyde, acrolein (the simplest, lowest homolog α,β-unsaturated aldehyde (Figure 1)) [64,65,66].

Therefore, the quantitative percentage of each of the above species generated is critically dependent on temperature, with the thermally induced oxidation at high frying temperatures favoring the degradation of lipid hydroperoxides to (*E*,*E*)-alka-2,4-decadienal (and smaller levels of its (*Z*,*E*)-isomer via a thermal isomerisation process [66]). However, autoxidation at ambient temperature favors the generation of hexanal. Complicating the picture is the fact that, as secondary aldehydes are further degraded, perhaps via retro-aldol condensations, the proportion of early-stage markers such as hexanal may diminish when expressed relative to alternative secondary LOPs [29,64]. Another layer of complexity comes from the fact that different foods are composed of different matrices of proteins, minerals, antioxidants, etc., which modify the rates of accumulation and degradation of these reactive LOPs [29,64].

### 2.2. Aldehydes from Omega-3 Alpha-Linolenic Acid (ALA)/Linolenoylglycerols

From the peroxidation of alpha-linolenate/linolenoylglycerols, lipid hydroperoxides are fragmented to form a very complex range of volatile secondary products, including aldehydes as major decomposition products. However, the final yield is dependent on the peroxidation environment (for example, temperature and the availability of oxygen and suitable catalysts). Typically, aldehydes comprise 60–70% of the total identified fraction of volatile species [66,67].

Major aldehydic LOP species generated from the peroxidation of linolenate/linolenoylglycerols comprise propanal, which is the most abundant saturated aldehyde, along with an unsaturated aldehyde pattern including (*E*)-2-pentenal and (*E*)-2-hexenal, acrolein, (*E*,*E*)-hepta-2,4-adienal and HHE, as noted above; however, as with linoleate/linoleoyglycerols, the latter 4-hydroxy-alkenal is formed in low yield only. Importantly, in regulated gas chromatography–mass spectrometry (GC-MS) headspace analyses for volatiles in samples of heated linolenic acid, the aldehydes formed typically comprise nearly 20 distinct species, and these are the most predominant of the volatile product profile when compared to those of other secondary LOPs, such as alcohols, ketones and furans [68]. Hence, low-molecular-mass *n*-alkanals such as propanal may serve as markers of thermo-oxidative damage to culinary oil linolenoylglycerols, as outlined in Ref. [69].

### 2.3. Acrolein

Acrolein has the molecular structure H_2_C=CH.CHO, is the smallest α,β-unsaturated aldehydic LOP generated, and perhaps the most reactive and toxic of them. As with many other aldehydes, food consumption is the chief pathway through which humans are exposed to this toxin. The food type and the forms of food processing give rise to diverse and differing levels of acrolein in food. Interestingly, oil-soluble acrolein levels in frying oils can be much higher than in the fried products (frying rapeseed oil, for example, contains up to around 3700 times more acrolein than that of fried doughnuts [60,61]). At least some acrolein is stripped away after its production in processed foods, probably from associations with other food constituents. Still, even after consuming these foods, people may be reintroduced to this eliminated form of acrolein. Indeed, previous studies conducted have shown that volunteers experienced up to a 38-fold increase in internal acrolein levels after eating potato crisps [61]. In this [61], and another [70] recent reports, the process of acrolein formation in foods, combined with both in vitro and in vivo transformation pathways associated with the toxicological mechanisms of acrolein, and its interactions with small molecules and biomacromolecules, are discussed in detail.

### 2.4. H NMR Analysis of Thermally Stressed Culinary Oils

Typical ^1^H NMR profiles of the aldehyde resonance patterns generated in a commercially available canola oil sample exposed to thermal stressing at a temperature of 180 °C for durations of up to 90 min are shown in Figure S1 of the Supplementary Information. Additionally, a ^1^H NMR evaluation of aldehydic LOPs found in a packeted potato crisp product is displayed in Figure S2 of this document; these experiments involved their direct extraction with the NMR solvent deuterated chloroform. ^1^H NMR analysis of a series of aldehydic LOPs derived from the thermal stressing of soybean, corn, avocado and extra virgin olive oils according to standard frying practices at 180 °C for a 90 min period are shown in Figure 2, whilst Figure 3 shows plots of the time-dependent generation of (*E*)-2-alkenals, (*E*,*E*)- and (*Z*,*E*)-alka-2,4-dienals, 4,5-epoxy-(*E*)-2-alkenals, and long-chain saturated *n*-alkanals from corn, extra-virgin olive and two different soybean oil products during heating at this temperature for durations of 0–90 min (4-hydroxy- and 4-hydroperoxy-(*E*)-2-alkenal LOPs were not analytically distinguishable from each other, but they were from all other aldehydes detectable). A guide to assignments of ^1^H NMR signals of aldehydic-CHO functions detectable in culinary oils is shown in Table S2.

As expected, PUFA-rich oils, notably corn oil, and also soybean oil to a lesser extent, produced the highest amounts of (*E*,*E*)- and (*Z*,*E*)-alka-2,4-dienals since they are sequentially derived from this source only. Moreover, oxidation-resistant MUFA-rich olive oils generated much lower amounts of all aldehydic LOPs, most notably those derived from PUFAs alone (approximately one-half that of PUFA-rich corn oil), especially the oxygenated α,β-unsaturated aldehydes. Also notable is the fact that 4-hydroxy-(*E*)-2-alkanals such as the extremely toxic HNE only arise from so-called “heart-friendly” PUFAs, and **not** MUFAs! Hence, the authors thoroughly recommend the future use of MUFA-rich oils such as olive oil for high-temperature frying or cooking practices. Notably, in accordance with previous publications [71,72], and also our summaries given above, the orders of the rate and extent of aldehyde generation for each class of aldehyde monitored are (*E*)-2-alkenals > (*E*,*E*)-alka-2,4-dienals > *n*-alkanals > 4,5-epoxy-(*E*)-2-alkenals ≈ 4-hydroxy-/4-hydroperoxy-(*E*)-2-alkenals when these are expressed as percentages of the total amount formed. However, that for (*Z*,*E*)-alka-2,4-dienals varied somewhat amongst the oils investigated, although it should be noted that it some of it presumably arises from the isomerism of its (*E*,*E*)-isomer [66], which, in turn, is derived from PUFAs only and not MUFAs.

The SFA, MUFA and PUFA contents (*w*/*w*) of the unheated (control) oils were: extra-virgin olive oil 17.0, 76.0 and 7.0% (*w*/*w*); avocado oil 16.0, 70.5 and 13.5% (*w*/*w*); corn oil 12.9, 27.6 and 59.5% (*w*/*w*); and soybean oil 17.7, 24.1 and 58.2% (*w*/*w*), the latter consisting of 50.7% linoleate and 7.5% linolenate.

Each 90 min heating cycle was conducted n = 6 times for all oils investigated. LSSFEs involved the heating of a 6.00 mL volume of culinary oil in a 250 mL thoroughly air-dried glass beaker maintained within a silicone oil bath accurately regulated at a temperature of 180 °C throughout the total 90 min. heating period. Aliquots (ca. 0.20 mL) of oil samples were collected at the above heating time-points for ^1^H NMR analysis. Samples were prepared for ^1^H NMR analysis, including treatment with the antioxidant *di*-*tertiary*butylhydroquinone (DTBHQ), as described in Figure S1.

### 2.5. Factors Affecting Dietary LOP Consumption by Humans

A major increase in exposure to LOPs occurred in countries that banned the use of partially hydrogenated oils, which was attributable to the era in 2004–2018 that saw the removal of partially hydrogenated oils from the food supply of many developed countries. In these countries, restaurants, hospitals, healthcare facilities, institutions, school cafeterias, bakeries, fast food, street food, and large-scale food manufacturers shifted almost entirely to high PUFA content cooking oils for frying and for processed food production. Hydrogenated cooking oils had been developed for use in commercial settings because the hydrogenation process improves oxidative stability by converting PUFAs into monounsaturated and saturated fats, which better resist oxidation. Unfortunately, many liquid cooking oils are considerably less stable under the same conditions because of their high PUFA content [58,67]. Toxicologists specializing in lipid chemistry have long recognized that the heating of PUFA-rich oils can cause their degradation, resulting in substantial increases in secondary oxidation product production, including that of α,β-unsaturated and less toxic saturated aldehydes [73,74,75,76]. This understanding is well established in the food science and edible-oil industries, although it is rarely communicated to clinicians or incorporated into dietary guidelines available for pregnancy.

PUFA oxidation products, including aldehydes, are already present in refined cooking oils in trace amounts before heating [48,73]. Air and light can also slowly convert PUFAs into LOPs. However, heat energy dramatically accelerates PUFA oxidation, as can pro-oxidative compounds in foods such as redox-active trace metal ions [77]. The reactive aldehydes so generated include acrolein, 4-hydroxynonenal (4-HNE) and 4-hydroxyhexanal (HHE), which are among the most electrophilic and biologically reactive products of lipid degradation, and their toxicity has been extensively documented [78,79,80]. However, it should be noted that the most predominant α,β-aldehydes generated are admixtures of (*E*)-2-alkenals and (*E*,*E*)-alka-2,4-dienals (65 molar % or more, i.e., 0.65 mole per mole of total lipid present; these percentage figures are only slightly different from the more familiar weight percentages). Total levels of hydroxy-(*E*)-2-alkenals produced rarely exceed 7–8 molar %, and this is obviously a very important consideration when weighing up the scientific literature available on toxic dietary LOPs.

The injudicious application of heat to refined high PUFA content oils has underappreciated consequences. Indeed, commercial frying practices often involve maintaining the oil at high temperatures for many hours or even days, which permits the accumulation of considerable amounts of reactive aldehydes. For context, a 100 g (3.5 oz) serving of fast-food French fries contains approximately 4 mg of reactive aldehydes, while smoking a pack of cigarettes delivers 2.4–9.2 mg [66]. Home cooking can produce similar concentrations [66,76], particularly during pan frying, which exposes oil to significantly more oxygen than commercial deep frying processes. Many packaged and prepared foods also contain thermally oxidized cooking oils, adding to our total aldehyde exposure. Because these products are common in the diets of women of childbearing age, aldehyde exposure during early and late pregnancy may be far more prevalent than is currently appreciated.

When considered together, these trends have created an environment in which aldehydes generated from refined cooking oils are now a routine part of many maternal diets. Yet these exposures remain largely unmeasured, unmonitored, and absent from prenatal nutritional frameworks. Recognizing that reactive aldehydes are now highly prevalent in our food supply is an essential first step toward allocating support to evaluate their potential impact on fetal development. This is particularly important given the existing evidence that they may interfere with the formation of critical cortical structures that depend on highly regulated developmental processes.

Notably, acrolein has been found in a wide range of foods, including baked or fried foods, fruits, vegetables and alcoholic beverages. However, it has been shown that acrolein levels are significantly modified during food processing and ingestion episodes, an example of which is that thermally stressed rapeseed oil has an acrolein content of ca. 150 mg/kg [61,81] (nearly 3 mmol/kg), but potato chips pre-fried in this oil have only a limited acrolein concentration [80]. However, interestingly, the level of acrolein-related metabolites excreted in urine following potato chip consumption strangely appeared to be substantially higher than the computed acrolein content of the consumed chips [56], and hence it appears that it may chemically react with nucleophilic potato chip molecules to form adducts which re-release this aldehyde in vivo. However, if alcoholic beverages are also considered, the daily acrolein intake can readily exceed 1 mg [57]; this value is far in excess of the tolerable daily intake value of 7.5 µg/kg bw/day set by the WHO; however, it is important to note that major health organizations in the USA, the UK and elsewhere all state that alcohol should be avoided completely during pregnancy. Hence, in view of the contrasting dietary habits of consumers, the correct exposure level for acrolein is not easily determined, and obviously may be underestimated if its beverage content is neglected, as it often is. Intriguingly, acrolein consumption can play an important role in driving human NCDs such as cardiovascular disease [82].

Therefore, in view of the sensitivity of such species to acid hydrolysis, e.g., glycation products derived from the reaction of HNE with lysine, it is likely that “free” HNE may be derived from this primary Schiff-base compound from dietary sources, and also some from the more common dietary protein lysyl residue adducts such as some of those present in the stomach, so that they may penetrate the systemic circulation as a “free” aldehyde rather than a biotransformed, relatively inactive form [83]. Similarly, reversible reactions of aldehydes with amino acids and compounds with other amine groups to form Michael addition products can also yield “free” aldehydes for uptake in this manner.

However, such processes may be blocked by (1) cyclisation and dehydration of Schiff base adducts, a process giving rise to 2-pentylpyrrole products (epitopes which are widely employed as biomarkers) [84], and (2) facilitation of intra- or inter-protein cross-links by HNE and other 4-hydroxy-(*E*)-2-alkenals [85].

### 2.6. Human Consumption of Different Aldehyde Classes Detectable and Determined in Fried Foods

If we assume that the major peroxidation products created thermally in frying oil are total (*E*)-2-alkenal, (*E*,*E*)-alka-2,4-dienal, and *n*-alkanal classifications, which are derived from peroxidation of linoleoylglycerols, i.e., (*E*)-2-octenal, (*E*,*E*)-deca-2,4-dienal and *n*-hexanal, then from Ref. [76], our estimation of these secondary LOPs in 30 g only mean UK French fry portions become human consumption levels of 495 ± 72, 569 ± 84 and 505 ± 72 µg, respectively, per day. From these values, we can see that the daily TTC limit for (*E*,*E*)-alka-2,4-dienals is certainly exceeded 5-fold or so, whereas that for (*E*)-2-alkenals, which we ‘upgraded’ to a class II aldehydic toxin, is not that far from its specified value. The consumption of the least reactive *n*-alkanal aldehydes (with the exception of acetaldehyde) does not appear to present any health threat using these limits and the fried food consumption level. The more toxic α,β-unsaturated compounds account for 68% of these consumable levels. For the typical ‘large’ serving size of 154 g, these amounts are 2.54 mg for (*E*)-2-octenal, 2.92 mg for (*E*,*E*)-deca-2,4-dienal, and 2.59 mg for *n*-hexanal.

Of course, these estimated intake levels would more than double if we used the proposed “worst-case scenario” French fry consumption rate of 60 g/day in the UK [86]. Moreover, if we consider the consumption of other fried foods (such as that in Spain, who have a mean total fried food intake rate of 138 g/day), then the “worst-case scenario” for French fries alone may be very large indeed. It is also worth mentioning that so far we have not taken into account the inhalation of cooking oil fumes rich in aldehydes (especially the ones from shallow frying and wok cooking).

Unfortunately, with the exception of acetaldehyde from ethanol metabolism, currently, there is only very limited data available on the blood plasma concentrations of aldehydes, including aldehydic LOPs, present in blood plasma, and such studies have largely been restricted to investigations of patients with cardiovascular diseases, e.g., congestive heart failure in Ref. [87]. Table 2 shows mean concentration ranges, or concentrations of mean values of aldehydes measured in the blood plasma of human subjects in one cardiac study, i.e., those measured in n = 8 normal left ventricle (LV) function control patients using a GC/MS technique. Corresponding values for these aldehydic LOPs in commercially available potato chip samples are also shown for the purpose of comparative evaluations. Hence, if we exclude dietary furfural, there appears to be some partial correlation between these blood plasma concentrations and the content of aldehydic LOPs present in fried foods. Moreover, the relatively high blood plasma concentration of HNE determined indicates that a high proportion of it arises from an in vivo source.

**Table 2:** Mean concentrations, or concentration ranges of mean values, of aldehydes monitored in the blood plasma of n = 8 normal left-ventricular (LV) function human participants by a GC/MS technique (extracted from Ref. [87]). This Table is adapted from Reference [28] with permission. Abbreviations: nd, not determined.

Aldehyde Class	Normal LV Function Human Control Participant Blood Plasma Levels (nmol/L)	French Fry Content (µmol/kg)
**Long-chain *n*-Alkanals (7)**	69–573	19–560
**Short-Chain *n*-Alkanals (1)**	67	nd
**(*E*)-2-Alkenals (4)**	106–527	0–430
**4-Hydroxy-(*E*)-2-alkenals (4)**	33–211	0.5–2.1 *
**(*E*,*E*)-Alka-2,4-dienals (2)**	152–180	0–443
**(*Z*,*E*)-Alka-2,4-dienals**	nd	0–60
**MDA**	96	0–6 **
**Furfural**	2450	nd

The bracketed numbers in the first (molecular classification) column refer to the number of different aldehyde homologues featured for each classification specified for the blood plasma samples analyzed. Also listed are the ranges of contents (µmol/kg) found for samples of French fry samples purchased from fast-food/take-out restaurants (long- and short-chain *n*-alkanals, (*E*)-2-alkenals, and (*E*,*E*)-alka-2,4-dienals were determined by our ^1^H NMR analysis approach, * but those for 4-hydroxy-(*E*)-2-alkenals are as documented in Csallany et al. [63] using HPLC and HPLC/MS methods (however, both 4-hydroxy-(*E*)-2-alkenals and furfural are also readily ^1^H-NMR-detectable and quantifiable). ** MDA was specifically determined by the method described in Claxson et al. [58], which involved a protocol featuring the reaction of thiobarbituric acid (TBA) with this dialdehyde to form a pink/red chromophoric derivative, but only subsequent to its relatively specific extraction into an aqueous medium (mean ± SD first extraction efficacy: 78 ± 2%).

Therefore, if we exclude dietary furfural, there appears to be some partial correlations between these blood plasma concentrations and the content of aldehydic LOPs present in fried foods. Moreover, the relatively high blood plasma concentration of HNE may indicate that more or it arises from an in vivo source.

Unfortunately, we have no clear data as to how well these blood plasma concentration level data may apply to the fetal brain. Indeed, currently there are no corresponding levels available for maternal blood plasma samples. Since such parameters are not currently available in the scientific literature, ideally, future studies should include essential information on dietary aldehydic LOP fractions, human plasma concentrations, and effective concentrations from any in vitro investigations.

Additionally, it should also be noted that the above TTC values (Table 1) were never tested for fetal protection. The only validation study related to TTC for pregnant women that we could identify is Laufersweiler et al. (2012) [88], which assessed 283 chemical agents against general reproductive/developmental end-points in animals. Of the LA/ALA-derived aldehydes discussed in this paper, only acrolein appears in that dataset, informed by a single two-generation rat study; the quantitatively dominant species we identify as the primary dietary concern—(*E*)-2-alkenals, (*E*,*E*)-alka-2,4-dienals, and additionally the lower-level oxygenated alkenal classes—are entirely absent from it, and no fetal brain outcomes were examined for any chemical included. So, while Section 2.6 shows dietary exposure may exceed adult-derived TTC values, whether or not staying under these values would actually protect the fetal brain is simply not known at present.

#### Currently Recommended Maternal (Pregnant/Lactating) Diets

The official nutritional recommendation for total fat content is identical for both maternal (pregnant/lactating) and normal (non-pregnant) diets, requiring 20% to 35% of total daily calories derivable from fat. While the overall quantity remains the same, a maternal diet requires a strict focus on the quality and type of fatty acids consumed to properly support fetal brain and retinal development. Indeed, in pregnancy and breastfeeding, long-chain PUFA consumption should be closely monitored by women. Docosahexaenoic acid (DHA) and eicosapentaenoic acid (EPA) are critical nutrients for the healthy development of the baby’s brain and are vital for brain development in the baby’s CNS and cognitive development. DHA is critical for brain growth; EPA is crucial for brain function and health and is the most important PUFA to be taken on board during brain development. Currently, pregnant and breastfeeding women are advised to eat 1–2 servings of oily fish per week, such as salmon, trout, and sardines, and fish should be consumed in proportion to their fat/lipid intake. Unfortunately, both these very highly unsaturated fish oil PUFAs are substantially susceptible to peroxidation and LOP generation [89], and it might be prudent to consider steaming fish to better preserve their health benefits.

Interestingly, the lipid peroxidation profiles of lipid emulsion products used for parenteral infusions used in newborn infants have been previously investigated [90]. These parenteral lipid emulsions were found to be susceptible to lipid oxidation, and that products derived therefrom can exert damage to human cells, a process giving rise to hemolysis [91]. Hence, it is possible that the adverse effects putatively exerted by these infusions may be induced by such peroxidative damage reactions in vivo.

Similarly, in 2004, Scopesi et al. [92] explored cholesterol oxidation products (COPs) in frequently used intravenous lipidic emulsions. However, the levels of these agents found were extremely low and at or even below the limit of detection of the analytical technique employed, although it should be noted that 5 α-epoxycholesterol concentration was greater than that of 7-ketocholesterol. Therefore, COPs do not appear to represent a complication with the application of intravenous lipid emulsions.

### 2.7. Neurodevelopmental Evidence for the Most Prevalent α,β-Unsaturated Aldehydes: (E)-2-Alkenals and (E,E)-Alka-2,4-Dienals

Although differences in relative potency were evident, all conjugated analogs tested were equiefficacious with respect to the maximal neurotoxicity achieved. In contrast, nonconjugated alkene or aldehyde congeners did not cause synaptosomal dysfunction or sulfhydryl loss. Acrolein and other alpha,beta-unsaturated carbonyls are bifunctional (electrophilic reactivity at the C-1 and C-3 positions) and could produce in vitro neurotoxicity by forming protein cross-links rather than thiol monoadducts. Immunoblot analysis detected slower-migrating, presumably derivatized, synaptosomal proteins only at very high acrolein concentrations (≥25 mmol/L).

Interestingly, in 2006, LoPachin et al. [93] examined structure–toxicity correlations for a range of conjugated type-2 alkenes, including acrolein and higher homologues (*E*)-2-alkenals, this serving as a means of elucidating the mechanism of the thiol adduct generation and associated neurotoxicological importance for these compounds. In rats, synaptosomes exposed to these compounds showed neurotoxic effects that were concentration-dependent and were associated with the loss of free sulfhydryl groups. Despite there being differences in the relative potency of all conjugated analogs tested, all of these were equiefficacious regarding the maximal level of neurotoxicity attained. Contrastingly, unconjugated alkene or aldehyde derivatives did not give rise to synaptosomal damage, nor the loss of sulfhydryl groups, as expected.

Acrolein and related α,β-unsaturated carbonyl compounds are, of course, bifunctional, exhibiting electrophilic reactivity at both C-1 and C-3 sites, and may cause in vitro neurotoxicity via the formation of protein cross-links, and not through thiol mono-adducts. However, hydralazine at a level of 1.00 mmol/L, which inhibits protein cross-link formation, did not affect the neurotoxicity caused by acrolein in vitro. Hence, type-2-conjugated alkenes were found to cause synaptosomal toxicity in relation to a corresponding decrease in thiol content, and this supports the hypothesis that the mechanism of acrolein neurotoxicity involves the formation of Michael adducts with protein sulfhydryl groups.

The above mechanisms also relate to the neurodegenerative actions of (*E*,*E*)-alka-2,4-dienals. However, these di-unsaturated aldehydic LOPs, which are important constituents of used cooking oil and their fumes, may drive neurodegeneration by triggering a rapid spurt of oxidative and endoplasmic reticulum stress (it diminishes mitochondrial membrane potential and intracellular ATP concentrations, and enhances ROS generation [94]). Moreover, these aldehydes also weaken metabolic detoxification and promote vascular damage. Since the brain is critically dependent on rigid glucose homeostasis and an intact vascular system, microvascular dysfunction caused by (*E*,*E*)-deca-2,4-dienal readily compromises the blood–brain barrier and exacerbates cognitive decline [95].

## 3. Dietary Aldehydes Are Absorbed and May Penetrate and Affect the Fetal Environment

To enter the fetal brain, aldehydes would need to (1) be absorbed into the mother’s gut, (2) be transferred through the placenta, and (3) pass through the fetal blood–brain barrier (BBB). To our knowledge, no study has yet attempted to evaluate these phenomena, which would probably require, for example, radiolabeling a specific dietary aldehyde and tracing its distribution through the fetal tissues. In the absence of such experiments, this section will discuss what the literature has revealed regarding absorption and distribution and passage into the brain, and separately consider passage through the placenta. Although we review the available evidence for each of the individual steps here in Section 3, it is important to remember that maternal dietary aldehydes need not cross either the placenta nor the fetal BBB to negatively impact neurodevelopment. The only requirement is that they are absorbed into the maternal circulation, because there they may promote the maternal “oxidative stress” that subsequently negatively impacts the developing fetal brain, which is the central concern of the current paper. Direct evidence for this maternal-to-fetal propagation of oxidative stress exists: maternal and cord (fetal) blood markers of lipid peroxidation and antioxidant enzyme activity are significantly correlated, with this relationship, becoming markedly more pronounced under conditions of maternal oxidative pathology such as pre-eclampsia [96,97,98]. This possibility that dietary aldehydes can induce maternal oxidative stress that, by itself (i.e., without dietary aldehydes needing to penetrate the fetal brain), leads to fetal brain aldehyde exposure, and deleterious developmental effects will be explored in the following sections.

### 3.1. Absorption, Distribution and Metabolism of Key Toxic Aldehydic LOPs In Vivo

Substantial evidence demonstrates that reactive aldehydes generated during dietary lipid peroxidation are absorbed and metabolized following ingestion. In a landmark study, Grootveld et al. (1998) [99] demonstrated that two major α,β-unsaturated aldehydes generated during the thermal degradation of PUFA-rich culinary oils, (*E*)-2-pentenal and (*E*)-2-nonenal, are absorbed in vivo and subsequently metabolized through glutathione-conjugation pathways, yielding characteristic urinary mercapturate metabolites. Similar findings were reported by Kaye (1973) [100], which demonstrated the formation of identifiable urinary conjugates following exposure to acrolein, one of the most reactive and toxic α,β-unsaturated aldehydes. Together, these studies provide direct evidence that dietary aldehydes are absorbed and metabolically processed rather than remaining confined to the gastrointestinal tract.

As noted above for acrolein, additional evidence indicates that further aldehydic LOPs may also be absorbed from protein-bound forms. McGirr et al. (1985) [101] identified urinary metabolites derived from malondialdehyde (MDA)-protein adducts following oral administration, supporting the concept that aldehydic LOPs survive digestion and contribute to the systemic aldehydic burden. Likewise, Awada et al. (2012) [102] demonstrated intestinal absorption and biological activity of HHE, providing further evidence that dietary LOPs can enter the body and participate in biologically relevant processes. Collectively, these findings established that dietary aldehydes and related LOPs can be absorbed, distributed, and metabolized in vivo, providing a mechanistic basis for considering them biologically relevant dietary toxicants.

Aldehyde metabolism is an important detoxification process in which these highly reactive and cytotoxic compounds are mainly oxidized into less toxic carboxylic acids via the aldehyde dehydrogenase (ALDH) superfamily of enzymes. Indeed, extensive aldehyde exposure leads to severe oxidative stress, DNA cross-linking and protein damage, and is therefore critical to protect cells and tissues [103,104]. The main metabolic pathway for aldehyde clearance is an NAD(P)^+^ oxidation, in which a harmless corresponding carboxylic acid (i.e., acetate from acetaldehyde, propionate from propanal, etc.) is generated [103,104]. Human cells utilize ALDH enzymes in different compartments that process different classes of aldehydes.

Firstly, ALDH2 acts on acetaldehyde and HNE in the mitochondrial matrix, and this is crucial for alcohol clearance. However, an inactive variant in 8% of humans can cause an “alcohol flush reaction” and may also enhance cancer risk. Secondly, ALDH1A1 metabolizes retinaldehyde and various aliphatic aldehydes in the cytosol; it is important for the synthesis of retinoic acid during embryogenesis and cellular development [103,104]. Finally, ALDH3A2 consumes long-chain fatty aldehydes within the endoplasmic reticulum/peroxisomes; deficiencies of this enzyme cause Sjögren–Larsson syndrome, which leads to severe ichthyosis and neurological defects.

When the oxidative capacity via ALDH is low or compromised, secondary mechanisms are involved in the reduction of aldehydes to primary alcohols by NADPH-dependent aldose/alkenal reductases.

Highly reactive α,β-unsaturated aldehydes (e.g., acrolein, (*E*)-2-alkenals, (*E*,*E*)-alaka-2,4-dienals or HNE) can biochemically react with glutathione (GSH) at their C3-position to diminish their electrophilic toxicity [104]. Subsequent to this thiol conjugation process, enzymatic reduction or oxidation of the aldehydic function occurs to generate primary alcohol or carboxylic acid anion derivatives. Moreover, O-glucuronide metabolites are also readily detectable in the urinary ^1^H NMR profiles of animals pre-dosed with selected (*E*)-2-alkenals [99].

### 3.2. Passage Through the Blood–Brain Barrier (BBB): Previous Investigations Exploring Aldehydic Load in Animal Brain

Contrary to the long-standing assumption that the fetal BBB is structurally immature and more permeable than the adult barrier, tight junctions at the cerebral endothelium and choroid plexus are functionally effective from the earliest stages of development studied [105]. However, the fetal barrier differs meaningfully from the adult system in ways relevant to LOP exposure: Cerebrospinal fluid turnover is substantially lower in the fetus, allowing greater accumulation of molecules that do cross by passive diffusion or via developmentally regulated transport routes. The choroid plexus, rather than the cerebral vasculature, is the dominant portal of entry into the developing brain, and the profile of efflux transporters present differs from the adult, with some (e.g., P-glycoprotein) absent until later in gestation [105]. Through these mechanisms, dietary-sourced LOPs and/or their metabolic precursors and products may gain access to, and accumulate within, the developing fetal nervous system. However, as noted, studies directly tracing dietary aldehydes from the mother’s diet to the fetal brain are technically challenging, and, to the best of our knowledge, have not yet been performed.

Regarding studies performed during post-natal life, animal research has indicated that ingestion of oxidized PUFA-rich oils or pre-formed LOPs (in our view, incorrectly known as oxLAMs for oxidized linoleic acid/linoleoylglycerol metabolites) leads to measurable increases in aldehyde-derived adducts in blood and multiple organs [106,107]. Nevertheless, in a single controlled study conducted to date, Ramsden et al. (2018) [108] showed that dietary exposure to corn oil, which is high in LA, significantly elevated omega-6 PUFAs and oxidized LA “metabolites” (oxLAMS), but decreased omega-3 PUFA levels, in mouse brain. However, dietary oxLAMs exerted only a very limited effect on oxidized neural tissue lipids, but did diminish both omega-6 and omega-3 fatty acids. Moreover, neither dietary LA nor oxLAMs altered HNE levels, and, hence, this study largely returned a null hypothesis result for brain aldehydic load, i.e., HNE and further LOPs were not found to increase therein.

However, this question should certainly remain open since (1) the diet employed in the Ref. [108] investigation contained “oxidized” LA adducts rather than more realistic intact culinary corn oil thermally stressed according to standard frying practices; (2) the animals used were adult and not fetal; and (3) steady-state feeding for a duration of 8 weeks may not sufficiently model peak postprandial exposure.

This investigation featured the thermal stressing of a commercial corn oil sample for up to ca. 4 weeks in a shallow cast iron pan at 115 °C. Solutions of this oil in deuterochloroform (CDCl_3_) were then analyzed using medium-field ^1^H NMR analysis (400 MHz operating frequency only). These researchers then state that they determined the amounts of PUFAs consumed during exposure to their heating episode by electronically integrating the oil’s *bis*-allylic-CH_2_ multiplet signals (δ = 2.76 ppm), and normalizing this intensity to those of the glyceryl-CH_2_OH protons located at δ = 4.13 and 4.28 ppm, which were not affected by oxidation. ^1^H NMR resonances arising from both *cis*,*trans*- and *trans*,*trans*- isomers of conjugated hydroperoxydienes (HpODEs) (δ = 5.40–5.55 ppm, 5.86–6.11 ppm, along with smaller multiplet resonances located at δ = 6.12–6.32 ppm) were reported, but it appears that no attempt was made to confirm the identities of and quantify these primary LOP species. Moreover, the authors of Ref. [108] also mention that minor aldehyde proton signals were found in the (rather limited) 9.40–9.56 ppm chemical shift range, but, again, this statement is very vague, with no attempt made to quantify the levels present in their heated corn oil test product. Since this is the case, the results acquired from Ref. [109] are certainly not relevant to our objectives in the current study, in which we predominantly consider and evaluate the fetal brain-disrupting effects of secondary aldehydic LOPs.

Based on the LA composition of corn oil as 55% (*w*/*w*) in the 2 kg sample of oxidized product employed in Ref. [108], according to the authors, there were 192 g of HpODEs present in the total oil mixture at the end of the thermo-oxidative reaction process, as calculated from the ^1^H NMR integration results (this does not make any sense from their oxidized lipid diet compositions provided below, however). Nevertheless, this again is very vague and uninformative, since these primary peroxidation products are very active precursors of many secondary and further LOPs. There is not even an estimate of the relative proportions of *cis*,*trans*- and *trans*,*trans*-isomers present, which is actually readily determined from ^1^H NMR profiles [48].

So, what was actually employed as a source of oxLAMS in Ref.’s [108] study? Certainly not aldehydic LOPs, nor their conjugated lipid hydroperoxide precursors. Firstly, the concentrations of oxLAMs present in the heated corn oil test matrix used by these researchers are only nmol/g, which is equivalent to only virtually trace µmol/kg levels. “Real-Life” values of aldehydes and their hydroperoxide precursors in PUFA-rich culinary oils such as corn oil exposed to standard frying practices (e.g., for 30 and/or 60 min at 180 °C) can easily exceed 10–20 mmol/kg for both groups, so the approach used by Ramsden et al. [108] certainly appears to be erroneous anyway. The oxLAMs present in what they describe as their “Low LA + OXLAM” diet were **9-hydroxy**-octadecadienoic acid (9-HODE), mean value only 20.0 µmol/kg; **13-hydroxy**-octadecadienoic acid (13-HODE), mean value only 67.5 µmol/kg; **9-oxo**-octadecadienoic acid (oxo-ODE), mean value only 3.6 µmol/kg; **13-oxo**-octadecadienoic acid (oxo-ODE), mean value only 1.9 µmol/kg; **9(10)-epoxy**-octadecenoic acid (EpOME), mean value only 51.8 µmol/kg; **12(13)-epoxy**-octadecenoic acid (EpOME), mean value only 109.3 µmol/kg; and **12,13-dihydroxy**-octadecenoic acid (DiHOME), mean value only 3.4 µmol/kg. Hence, neither PUFA-derived hydroperoxides (isomeric CHPDs) nor their aldehydic fragmentation products (Figure 1) are mentioned at all in this list, so we can only assume that, despite their ^1^H NMR analysis, they were not further considered by the authors. What happened to them? Hence, this account confirms that results acquired from that study are not at all relevant to our hypotheses in the current paper.

It may be useful to repeat these experiments with a more realistic model of thermal stressing and more attention paid to the aldehyde and CHPD precursor contents of a PUFA-containing or PUFA-rich culinary oil sample, since the approach adopted was unrealistic in terms of both the oil fed to the animals and the oxidation products measured. We point this out not as a criticism, but to illustrate the urgent requirement for better cross-disciplinary communication to improve overall experimental design.

### 3.3. Passage Through the Placenta: First-Pass and Placental Metabolism, and the Effect of Efflux Transporters on Aldehyde Concentrations in the Brain

The human placenta acts as a metabolic and physical barrier and is also observed to act as a “placental first-pass effect” to protect the fetus against attack from reactive aldehydes and further LOPs. This protection is supported by appropriate combinations of metabolic enzymes and ATP-Binding Cassette (ABC) efflux transporters [109,110].

For the first-pass and placental metabolism of aldehydes, such species are very highly reactive, genotoxic molecules that may arise from the direct maternal intake of LOPs (also including the degradation of ethanol to acetaldehyde), internal cellular processes (in vivo lipid peroxidation) or embryonic metabolism. Indeed, in the first-pass mechanism, the outer part of the placenta, the syncytiotrophoblast, accumulates lipid-soluble aldehydes before they can reach the umbilical vein. The placenta achieves this without assistance via the use of special Phase I metabolic enzymes, which also feature the mitochondrial oxidation of acetaldehyde to acetate [110,111].

Protein expression of mitochondrial acetaldehyde dehydrogenase 2 (ALDH2) occurs as a first defense mechanism of the fetus and facilitates reductions of toxic aldehydes in the blood and cell membranes. However, greater than 80% of total placental ALDH activity is present in the cytosol (predominantly high-Km cytosolic ALDH variants). Although it is of much importance to control “surge” levels, its low basal affinity gives rise to the rapid release of high maternal levels of aldehydes (such as those produced by heavy alcohol consumption) into the fetal system [112,113,114].

However, efflux transporters can also exert major effects on aldehyde metabolism; indeed, placental ABC efflux transporters do not directly degrade aldehydes, but they can influence the xenobiotic’s intracellular residence time and substrate level [109]. Indeed, with regard to this residence time, the enhanced efflux clears parent compounds and circumvents rapid influx, and in this manner aldehyde levels remain low, and internal ALDH enzymes are never overwhelmed or saturated [109].

The influence of intracellular residence time enhancement on efflux pumps on the maternal-facing apical membrane, namely P-glycoprotein (P-gp/ABCB1) and Breast Cancer Resistance Protein (BCRP/ABCG2), complex xenobiotic aldehydes (or, where relevant, pre-aldehyde parent drugs) out of the trophoblast and back into maternal blood. By clearing parent compounds and preventing rapid influx, these transporters are able to maintain intracellular aldehydes at low levels.

When aldehydes are conjugated in phase II of metabolism (e.g., as glutathione, sulfate and glucuronide conjugates), they become highly anionic, and transporters comprising the Multidrug Resistance-Associated Protein (MRP/ABCC) family shuttle these conjugated metabolites out of the placenta [115]. Indeed, MRP2 transports neutralized metabolites back into maternal blood. MRP1 on the basolateral membrane can then pump them into fetal circulation for renal clearance by the fetus.

In view of spatial vulnerabilities and gestational shifts, transporters and metabolic routes interact very differently at the different stages of pregnancy. Indeed, during the first trimester, P-gp and BCRP expression are highest in early organogenesis, and this, in turn, creates a very tight gatekeeping mechanism to maintain external “foreign” aldehyde concentrations low so that the young placental ALDH2 can effectively process further incoming toxins [116]. Hence, P-gp and BCRP expression mediates the effective efflux of toxic xenobiotics such as aldehydes across cell membranes. P-gp and BCRP are commonly co-expressed alongside aldehyde dehydrogenases [115].

However, throughout the second trimester, the placenta is much more dependent on its own internal metabolism to manage the growing metabolic waste and aldehyde burden of the growing fetus (as the P-gp and BCRP expression naturally degrades towards term) [117]. Notably, a number of conditions can influence ABC transporter expression, and when these transporters are downregulated, aldehydes can pool in the trophoblast, which gives rise to marked oxidative stress, cell apoptosis and restricted fetal growth [117,118].

Along with dietary aldehyde consumption and transfer, maternal oxidative stress may trigger lipid peroxidation within the placenta or fetus. Indeed, reactive aldehydes may have the capacity to physically alter placental–brain axis gene expression, a process accumulating in lipid-rich fetal brain tissue and impacting neurogenesis. However, recently, Rasool et al. [119] found that exposure to the oxygenated aldehydic LOPs, HNE and HHE, differentially impacted lipogenic pathways in human placenta, and these effects may indeed have ramifications for the relative efficacies of omega-3 FA supplementations in loci featuring oxidative stress.

Hence, this marked body of evidence serves to support the existence of well-developed and highly evolved complex barrier mechanisms in the developing brain, which should be contrasted with the common belief of neurotoxicologists that the blood–brain barrier is immature or even non-existent in the embryo and newborn. Therefore, a proper and complete understanding of how such barriers work to prevent the entry of harmful substances or therapeutics into the developing brain is of critical importance for further research conducted in this area.

## 4. Brief Summary of Chemical Reactivity of LOPs with Endogenous Biomolecules Which May Trigger Human Diseases

LOPs react with biomolecules to produce characteristic products and, from these, further reactive compounds that could lead to oxidative stress, along with accompanying cell and tissue damage. These LOPs can covalently react with and bond to proteins, polysaccharides, DNA and phospholipids [59,65], and such products exhibit a multitude of physiological and pathological mechanisms in vivo. Indeed, they form stable Schiff bases with amino acids, especially lysine, cysteine, and histidine, together with other nucleophilic amino acids. The production of these Schiff base species is reversible in aqueous solutions, however. LOP interactions with biomolecules are a core area of great research interest, since they have a very broad significance for both health and clinical sciences. In addition, unsaturated aldehydes such as the different classes of α,β-unsaturated aldehydes are able to readily engage in Michael addition reactions with glutathione (GSH) to form primary detoxification GSH conjugate products at the C3-position, but with *n*-alkanals, they may only form Schiff base adducts with the terminal amino group of this tripeptide. Indeed, the relative concentrations of the infamous two “***scientifically overattended***” aldehydes HNE and MDA indicate—but certainly do not establish to an absolute degree—that such toxins possibly resulted from lipid peroxidation in vivo [120]. This result is consistent with the idea that human MDA is also produced via frying episodes conducted at high temperatures, as well as by the prolonged storage of foods rich in its PUFA precursors, as we might perceive.

In spite of the established ability of lipid peroxidation-derived aldehydes to further perpetuate oxidative stress in living systems, in the late 1990s and early 2000s, the observation that these reactants, such as HNE, modify specific proteins and specific residues, rather than reacting uniformly across the proteome, was widely interpreted as evidence against a purely “random” toxicological role. This interpretation led many to discount the toxicity of these molecules and view them as helpful regulatory molecules with important signaling functions [121]. This interpretation gained further traction with the characterization of KEAP1 as a cysteine-based electrophile sensor, the modification of which activates NRF2-driven antioxidant gene expression [121], after which much of the literature shifted focus towards the adaptive and cytoprotective potential of these aldehydes, with comparatively less attention paid to their capacity for direct molecular damage. However, the observed selective targeting may be explained by other variables, and this does not serve as evidence that these molecules are benign. The existence of a dedicated cellular sensor for these compounds indicates only that the body has evolved specifically to detect and defend against the damage that specific aldehydes such as HNE cause. It therefore remains important to consider aldehydes as toxicants capable of causing direct harm, particularly when a dietary source, such as high-PUFA culinary oils, may add to the body’s existing endogenous aldehyde burden and may thereby contribute to disease. In our view, all proteins with accessible reactive thiol (-SH) functions in vivo may react with and at least partially neutralize α,β-unsaturated aldehydes via Michael addition processes. Table 3 presents a representative overview of the capacity for only three of the most well-studied aldehydes to electrophilically attack proteins and disrupt their function.

## 5. Dietary LOPs and Aldehydes May Disrupt Fetal Brain Development

### 5.1. Brain Development and Function Is Highly Dependent on Normal Cytoskeletal Protein Dynamics

Major cytoskeletal proteins include actin, spectrin, tubulin, and vimentin. These proteins, and others, work together to support numerous neural functions that are vital to cell survival. For a nerve cell to function normally, these proteins must be able to constantly interact, and this motion forms the basis for all human (and animal) thought.

Disruption of cytoskeletal dynamics at different points in brain development is expected to produce different specific phenotypes, but interference with any of these sequentially dependent processes converges on abnormal brain structure and disrupted function. For example, normal sensory processing depends on the formation of a six-layered neocortical architecture between weeks 6–22, and disruptions of this architecture have been implicated in a broad range of neurodevelopmental disorders characterized by altered sensory processing, cognition, behavior, and motor function [124,125,126,127]. Conditions such as autism spectrum disorders, attention-deficit/hyperactivity disorder (ADHD), schizophrenia, and developmental dyslexia are well-known neurodevelopmental and neuropsychiatric disorders that arise in part from altered neural network structure. Thus, an exploration of LOPs and their interactions with cytoskeletal proteins, as well as their impact on redox balance in vivo, is warranted.

### 5.2. Cytoskeletal Integrity Is Particularly Vulnerable to Aldehydic Modification

In view of their chemical reactivity, α,β-unsaturated aldehydes generated during lipid peroxidation preferentially target a small sub-set of nucleophilic amino acid side-chains, predominantly cysteine, histidine, and lysine residues, and, to a lesser extent, arginine, with Michael addition adduct formation following a consistent order of reactivity: cysteine > histidine > lysine > arginine [123,128]. As shown in Table 3, lipid-derived aldehydes target at least seven distinct cytoskeletal proteins, as well as collapsin regulatory protein 2, which plays a role in axon specification and growth cone extension, providing a potential mechanistic link between dietary LOP/aldehyde exposure, cytoskeletal dysfunction, and altered brain development. Animal experimentation provides support for the link, as discussed below.

### 5.3. Aldehyde-Related Cytoskeletal Disruption Can Affect Multiple Stages of Cortical Assembly

In vitro experimentation on animal neuronal tissue has demonstrated that cooking oil-derived aldehydes modify cytoskeletal proteins required for both normal neuronal process formation and the subsequent development of cortical circuits. Neely et al. [129] demonstrated that HNE modification of tubulin disrupted neuronal microtubules and inhibited neurite outgrowth. Huang et al. [130] showed that acrolein exposure altered multiple aspects of neurite physiology, a process giving rise to reduced neurite extension, neuritic beading, and discontinuity of neuronal processes. Similarly, Lovel et al. [131] reported neuritic degeneration, neuronal process injury, and cytoskeletal damage following acrolein exposure to cultured hippocampal neurons. These are just a few specific examples. Others have shown that disruptions to cytoskeletal maintenance or development are associated with a range of disruptions to learning and memory formation, and are involved in numerous neurodevelopmental diseases, including everything from subtle learning to autism spectrum disorders, along with congenital anomalies [132,133,134].

Radial glia depend on intact intermediate filaments, membrane adhesion complexes, and mitochondrial ATP production in order to maintain their elongated scaffold and support neuronal migration. Aldehydes modify and cross-link vimentin, glial fibrillary acidic protein (GFAP), and adhesion molecules such as neural cell adhesion molecule (NCAM) and L1 cell adhesion molecule (L1CAM), a process compromising scaffold stability [135,136]. In addition, aldehyde-induced mitochondrial dysfunction reduces ATP availability and impairs Ca^2+^-dependent cytoskeletal remodeling. These combined effects promote migration errors and focal malformations of cortical development (MCDs), many of which have been documented in developmental disorders involving abnormal cortical organization and connectivity [137,138,139,140].

### 5.4. Aldehydes Promote Oxidative Stress and Can Amplify These Developmental Defects

Aldehydes create a pro-oxidative environment by forming adducts, primarily with glutathione through Michael addition or other reactions, and inhibiting antioxidant enzymes, including glutathione peroxidase, glutathione-S-transferase, and thioredoxin reductase. Indeed, oxidative stress alone is sufficient to disrupt neuronal migration, induce premature differentiation, and alter laminar thickness [138,139,140], and microtubules that support the cerebral cortex’s tightly-packed microcolumnar assemblies [141]. Because aldehydes both arise from and further propagate oxidative stress, other nutritional defects in the maternal diet may be expected to magnify these deleterious effects. For example, micronutrients such as zinc ions, iron ions and folate are essential for antioxidant function [142]. Moreover, deficiencies of these nutrients amongst women of childbearing age are unfortunately widespread throughout the world. For example, a 2022 report revealed that deficiencies in either iron or zinc ions or folate range from 48% in high-income countries to 80% in sub-Saharan Africa [143]. This is particularly concerning, given that people who eat more of the foods that are likely to be contaminated with the highest proportion of aldehydes and other LOPs, i.e., take-out foods and packaged foods, are the most likely to be deficient in such micronutrients [144].

Altogether, these mechanistic findings indicate that aldehydes may target core biological processes that construct the architecture required for normal, healthy brain function. The fact that aldehydes disrupt the same developmental pathways that produce deficits in sensory processing, cognition, social behavior, and motor function when perturbed in animal models provides a compelling rationale to investigate aldehydic load as a potentially modifiable contributor to altered cortical development in humans.

### 5.5. Teratogenic Properties of Thermally Stressed Culinary Oils

Aldehydic LOPs induce DNA and chromosomal damage during embryonic development, a period in which cell division and differentiation are extremely active—including those within the neural tube, the embryonic structure from which the entire central nervous system (brain and spinal cord) arises. In 2002, these facts prompted Indart et al. [145] to examine the teratogenic effects of thermally stressed safflower oil administered orally to pregnant rats, and whether or not this effect could be mitigated by the addition of the antioxidant alpha-tocopherol (alpha-TOH). Safflower oil was heated for 20 min using standard frying practices and then stored at −20 °C under N_2_.

Four groups of pregnant Wistar rats were used in this study. Two groups received 0.30 mL of heated oil (Group HO), and one of them received 150 mg of alpha-TOH (Group HOE). The other two were used as controls: one group received non-heated oil (O), and the other received no treatment (C). The oil was administered daily through gavage from day 1 to day 11.5 of gestation, and the animals were then euthanized for embryo examination. Both aldehydic LOPs and alpha-TOH were determined in the heated and non-heated oils. Malformation was scored specifically as a failure of the neural tube and surrounding structures—including brain sphere morphology, neural tube size, closure, and axial curvature—to conform to expected day-11.5 morphology.

The heating episode performed significantly increased the concentration of aldehydic LOPs in the oil (3.20 ± 0.54, 2.39 ± 0.22, 0.49 ± 0.02 and 1.13 ± 0.32 mmol/L for (*E*)-2-alkenals, (*E*,*E*)-alka-2,4-dienals, (*Z*,*E*)-alka-2,4-dienals and n-alkanals, respectively (mean ± SEM)) and decreased the alpha-TOH content (from 1.68 ± 0.08 to 0.65 ± 0.16 mmol/L). The malformation rate in the HO group was 21.73%, compared to 5.6% in the O group and 7% in the C group. However, when pre-heated oil was taken with accompanying alpha-TOH, the malformation rate dropped to 7%. These results indicate that millimolar levels of LOPs in heated cooking oils (unsaturated and/or saturated aldehydes) can exert strong teratogenic effects on neural tube closure in this animal model.

This study evaluated neural tube closure at day 11.5, which is equivalent to roughly days 21–28 of human gestation—the earliest patterning stage of the CNS, upon which all subsequent development (brain vesicle formation, cortical neurogenesis, migration, and lamination) depends. Disruption at this stage is therefore particularly consequential, representing failure at the structural foundation of the CNS. Similar exposures later in gestation would be expected to have different but nevertheless detrimental effects; however, to our knowledge, no follow-up studies have been conducted to evaluate this possibility.

## 6. Why Have Aldehydes Been Overlooked in Prenatal Nutrition and Neurodevelopment Research?

Despite decades of work conducted on oxidative stress, environmental toxicology, and maternal nutrition, aldehydes generated from the oxidation of dietary unsaturated fatty acids remain almost entirely absent from prenatal neurodevelopmental frameworks. One notable exception to this pattern is the field of total parenteral nutrition (TPN). TPN is administered to some of the most vulnerable patients in medicine, including premature infants, critically ill neonates, and pregnant women unable to tolerate enteral feeding. Historically, intravenous lipid emulsions relied almost exclusively on soybean oil rich in PUFAs and are therefore highly susceptible to peroxidation and the formation of reactive aldehydes [146,147]. As evidence accumulated that these PUFA-rich formulations likely contributed to poor neonatal outcomes, the field responded by reducing the proportion of soybean oil [148,149]. Medium-chain triacylglycerol (MCT) oil became a central component of newer-generation lipid emulsions (alongside olive oil and fish oil) precisely because of its high content of oxidatively stable saturated fatty acids [147,148]. This shift illustrates that when lipid oxidation chemistry and clinical nutrition have the opportunity for a direct dialogue, the response has been a deliberate, evidence-based reduction in adverse PUFA oxidation—raising the question of why an analogous recalibration has not yet occurred in dietary and prenatal nutrition guidance more broadly.

This longstanding failure to recalibrate is a result of multiple, easily correctable omissions. They include technical limitations in the analytical approaches most commonly used to study lipid peroxidation products, and partly from siloization: investigators in nutrition science, edible oil chemistry, toxicology, and developmental neurobiology rarely interact, allowing critical “blind spots” and unchallenged assumptions to persist, as summarized below.

### 6.1. Technical Limitations Underestimate Aldehyde Prevalence

Most biological investigations rely on targeted analytical approaches for LOPs, such as high-performance liquid chromatography (HPLC), enzyme-linked immunosorbent assays (ELISAs), immunochemical detection of aldehyde-protein adducts, targeted mass spectrometric methods, or, alternatively, the very poor, unspecific and limited thiobarbituric acid-reactive substances (TBARS) assay systems available for MDA. As a result, both MDA and HNE have become the most commonly measured and reported aldehydic LOPs. While these compounds are biologically relevant, their widespread use as biomarkers has created the impression that they represent the principal or even only aldehydes generated during lipid peroxidation. This is certainly not the case. There are potentially 100 or more of these, each one belonging to up to 15 or so different classes! Hence, to overcome these limitations, our group has employed high-resolution ^1^H NMR spectroscopy, a multicomponent analytical technique capable of simultaneously detecting and quantifying numerous classes of aldehydic LOPs within a single sample with minimal sample pre-treatment, as noted above.

Our investigations demonstrate that HNE accounts for only a small fraction of the aldehydes generated during the thermal oxidation of PUFA- and linoleoylglycerol-rich culinary oils, as does MDA [28]. Although MDA typically represents approximately < 5 molar % of secondary aldehydic LOPs, and HNE approximately 5% or less, high-resolution ^1^H NMR analyses have revealed substantially larger quantities of other aldehydes, as noted above in Section 2 for soybean oil heated according to standard frying practices for a 90 min period. However, commonly encountered ranges for these mean molar percentage aldehyde concentrations include those for (*E*)-2-alkenals (approximately 30–42% (*w*/*w*) of total aldehydic products), (*E*,*E*)-alka-2,4-dienals (20–35%), (*Z*,*E*)-alka-2,4-dienals (8–17%), and *n*-alkanals (15–30%) [66,95]. Figures for the oxygenated α,β-unsaturated aldehydes are relatively low, with the 4,5-epoxy-(*E*)-2-alkenals and combined 4-hydroxy-/4-hydroperoxy-(*E*)-2-alkenals amounting to < 9 and < 7% of the total aldehydic LOPs generated, respectively. Notably, 4-hydroxy-(*E*)-2-alkenals are directly derived from the reduction of their corresponding 4-hydroperoxy derivatives, whereas the very highly toxic 4,5-epoxy-(*E*)-2-alkenals arise from the thermal decomposition of corresponding (*E*,*E*)-alka-2,4-dienals [66]. Consequently, studies that measure only HNE and/or MDA may substantially underestimate total aldehydic exposure arising from oxidized dietary lipids. This methodological limitation may have contributed to the broader under-recognition of dietary aldehydes as biologically relevant exposures in pregnancy and fetal neurodevelopment, or in other clinical areas which may be affected by the consumption of dietary LOPs.

### 6.2. Interdisciplinary Siloization Fosters “Blind Spots” and Unchallenged Assumptions

Within the edible oils and food processing literature, aldehydes generated during high-temperature treatments applied to PUFA-rich oils are considered important because of their associations with rancidity and other deleterious sensory effects. Within this context, they are classified as process contaminants, and in view of the lack of collaboration between scientists working for the processed food industry and scientists investigating the effects of process contaminants on human health, their ultimate biological effects are not often considered or are even ignored completely. Furthermore, the very same aldehydes that develop in these industrial and commercial settings also form during routine home cooking, where no analogous terminology exists. This linguistic gap reflects a broader conceptual gap: without a descriptive noun, these exposures cannot easily be studied, quantified, or incorporated into prenatal dietary frameworks. A uniform descriptor (e.g., “cooking oil-generated aldehydic toxicants”) may help close this conceptual gap and facilitate such interdisciplinary study.

Prenatal nutrition research traditionally focuses on micronutrient sufficiency (folate, choline, iron ions), essential FAs, glucose regulation, and maternal metabolic status. With little collaboration between perinatologists and lipid oxidation experts, dietary fats are generally evaluated mainly according to their effects on serum cholesterol levels, rather than on their ability to support a developing fetus. Their oxidative stability or their capacity to generate reactive lipid-derived electrophiles when heated is certainly not commonly discussed, if ever.

Clinicians caring for pregnant women may not realize that refined PUFA-rich cooking oils have rarely been evaluated as independent variables in view of the tendency to group foods, particularly refined cooking oils, into broad dietary categories such as “vegetable oils,” “polyunsaturated fats,” or “heart-healthy eating patterns” [97,98]. This categorical grouping produces a nutritional halo effect: cooking oils inherit the apparent benefits of foods such as nuts, seeds, fish, fruits, and vegetables that accompany them in these patterns, making it nearly impossible to detect any adverse effects uniquely attributable to oxidized oils or their aldehydic breakdown products. In 2007, Jacobs & Tapsell [150] pointed out that when foods appear only within broad “healthy patterns,” their specific biological effects cannot be isolated”. Moreover, since dietary pattern analysis evaluates the eating pattern as a whole, it cannot isolate the health effects of any specific nutrient, including culinary oils, as noted by Hu in 2002 [151]. Indeed, our Research Group [28,29] warns that this is likely problematic since the oxidative susceptibilities of different culinary oils differ profoundly; however, these differences are very rarely considered in nutritional assessments or dietary guidelines. Consequently, the well-established transformation of PUFA-rich cooking oils into reactive aldehydic oxidation products—long recognized in lipid chemistry—has not been incorporated into dietary risk assessments for pregnancy.

The biological effects of oxidized dietary lipids are more widely known to animal nutritionists. Animal-feeding studies involving the employment of recycled, used cooking oils as an energy source, and now dating back several decades, have reported reduced growth performance, impaired feed efficiency, increased oxidative stress, and other adverse outcomes following consumption of oxidized fats and oils [152]. These observations have led animal nutritionists to treat lipid oxidation as a significant feed-quality concern, and have contributed to the development of modern feed-safety standards which limit or prohibit the use of recycled fats and oils in animal feed.

For its part, toxicology treats α,β-unsaturated aldehydes primarily as environmental pollutants (e.g., cigarette smoke, air pollution, industrial solvents) rather than as dietary exposures arising from frequent everyday cooking episodes. Although the cytoskeletal, mitochondrial, and signaling toxicities of aldehydes are well documented, these findings have rarely been applied to questions of maternal–fetal nutrition, nor to fetal brain development.

Developmental neurobiology forms the third component of this “blind spot”. Research in this field is highly advanced in mapping the mechanisms of cortical lamination and neuronal migration, but it typically focuses on genetic perturbations and a narrow set of recognized environmental exposures such as valproate, ethanol, thalidomide, or maternal immune activation. Because aldehydes have not been considered relevant prenatal exposures, their potential capacity to disrupt the cellular machinery underlying corticogenesis has been almost entirely overlooked.

Taken together, these disciplinary silos have produced a three-component “blind spot”:

**1.**  **Nutrition science** does not evaluate aldehydes as maternal dietary risk factors;
**2.**  **Toxicology** does not commonly use the correct analytical equipment required to study the full spectrum of dietary aldehydes, nor does the discipline generally study dietary aldehydes in prenatal contexts;
**3.**  **Developmental neurobiology** does not account for diet-derived electrophiles that target the mechanisms of cortical assembly.

A systematic integration of insights from nutrition science, lipid oxidation chemistry, toxicology, and developmental neurobiology is now necessary in order to evaluate aldehydes as prenatal neurotoxicants. Without such integration, a major modifiable dietary exposure will continue to fall outside prenatal risk assessment. Closing this gap is essential for determining whether aldehyde exposure during pregnancy contributes towards altered cortical development and adverse neurobehavioral outcomes.

## 7. Identifying and Addressing Structural Barriers to Progress

Addressing the long-standing neglect of oil-derived aldehydes in prenatal neurodevelopmental research will require addressing structural barriers that prevent effective cross-disciplinary work. Scientists in nutrition, lipid oxidation chemistry, toxicology, and developmental neurobiology operate in separate intellectual ecosystems, with distinct terminology, experimental models, and funding pathways. No single discipline currently contains the conceptual tools required to link dietary or environmental aldehyde exposure to disruptions in cortical assembly.

Unfortunately, journal scopes and grant mechanisms reinforce this separation: nutrition studies rarely measure electrophilic LOPs; toxicology studies seldom incorporate developmental end-points; and neurodevelopmental research rarely includes dietary exposures as mechanistic variables. As a result, a biologically plausible class of prenatal toxicant has remained outside all major risk-assessment frameworks.

**Figure 4:**
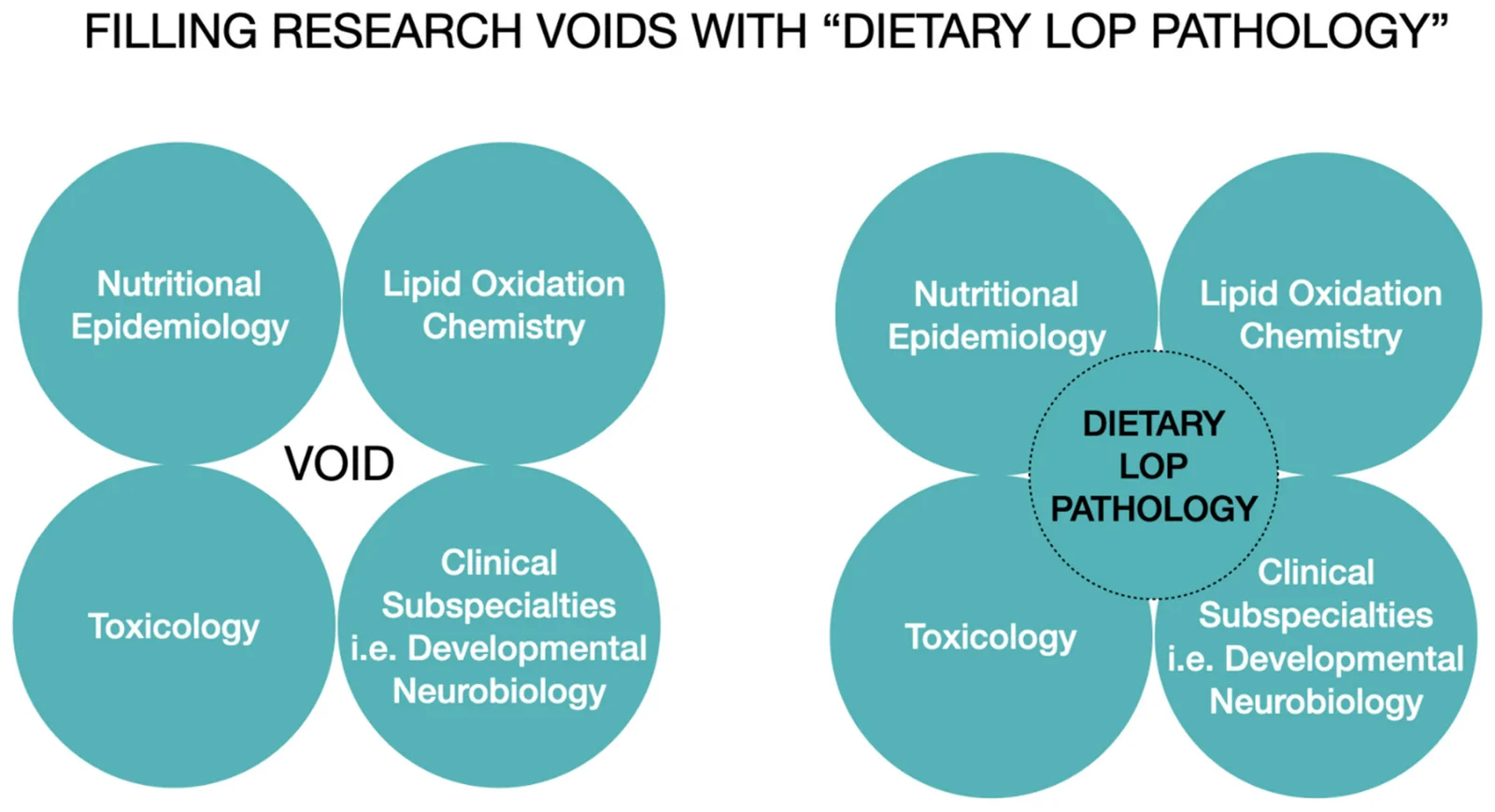
The left panel of this schematic illustrates how disciplinary fragmentation has limited investigation of lipid oxidation products (LOPs) derived from refined, high PUFA content culinary oils as prenatal exposures. Each field—nutritional epidemiology, lipid oxidation chemistry, toxicology, and clinical sub-specialties such as developmental neurobiology—addresses a distinct component of the pathway linking maternal diet to fetal neurodevelopment; however, these domains typically operate in isolation. The absence of integration creates a central gap in which no field captures the full trajectory from dietary exposure to molecular mechanism to developmental outcome. The (**right**) panel, however, depicts the conceptual closure of this gap through the introduction of an integrative framework, referred to here as ***Dietary LOP Pathology***. This domain centers on the role of diet-derived electrophilic compounds in disrupting cellular redox balance and influencing tissue development. By bridging these disciplines, such a framework may enable more comprehensive evaluation of aldehyde exposure as a modifiable factor in fetal neurodevelopment.

Closing this gap will require intentional collaboration across these domains, shared methodological standards for measuring aldehydes and aldehydic adducts, and project foci with funding mechanisms that prioritize integrative research capable of tracing dietary exposure through molecular mechanism to neurodevelopmental outcome. Ultimately, the developmental impact of lipid-derived aldehydes highlights the requirement for a new integrative domain within ***pathology***, to be known as ***Dietary LOP Pathology***, which could focus on how oxidative stress and electrophilic aldehydic LOP toxicants reshape cellular structure and function across organ systems. Such a framework could transform how we investigate dietary-derived toxicants, maternal–fetal exposures, and the redox-dependent mechanisms that sculpt the developing brain (Figure 4).

## 8. Research Priorities: Animal Experiments and Human Studies

The prevalence of neurodevelopmental disorders is on the rise, as is the attention being paid to them. Along with these trends, recent public health initiatives have emphasized identifying potentially modifiable environmental contributors to the rising prevalence of neurodevelopmental disorders. Further research into the link between cooking oil-derived aldehydes and altered fetal brain development could start with a systematic approach that prioritizes two complementary domains: animal experiments, which can establish causality and mechanism, and human studies, which can determine relevance at the population level.

### 8.1. Animal Experiments

Animal models offer the most direct means of testing whether high-PUFA cooking oils or aldehydic lipid-peroxidation products impair cortical development. Several high-value experimental strategies include:

#### 8.1.1. Controlled Feeding Studies Using Appropriate Animal Models, and High-PUFA Culinary Oils or “Real-World” Fried Foods

Current toxicological studies typically rely on purified aldehydes or oxidized linoleic acid (or linoleoylglycerol), and their metabolites. A critical next step is to test:

- Repeatedly heated PUFA-rich cooking oils;
- Fried foods at feeding levels representative of human exposure (e.g., chips, crackers, patties, French fries, etc.).

These interventions can be compared against fresh, unheated oils and saturated fat-rich controls to determine whether dietary aldehydes specifically drive neurodevelopmental abnormalities.

#### 8.1.2. Quantifying Aldehyde Load in Maternal and Fetal Tissues

Comprehensive aldehydic LOP profiling using high-resolution ^1^H NMR analysis, complemented by mass spectrometric determinations of aldehyde-protein and aldehyde-glutathione adducts, should be used to measure:

- Aldehyde accumulation in maternal plasma, placenta, fetal brain, and fetal liver;
- Glutathione depletion and oxidative stress markers;
- Aldehyde-protein adducts in neuronal and glial populations.

This would directly determine fetal exposure intensity and identify developmental “windows” of vulnerability. Further multi-analytical techniques should also be employed for these investigations.

#### 8.1.3. Assessing Structural Outcomes in the Fetal and Neonatal Brain

Experiments should evaluate whether high-PUFA oil exposure induces abnormalities consistent with known neurodevelopmental pathologies, for example, autism spectrum-related neuropathology, which demonstrates:

- Disrupted radial migration;
- Lamination defects;
- Altered minicolumn width or spacing;
- Focal cortical dysplasia.

These can be assessed using Nissl staining, immunohistochemical layer markers, diffusion-weighted MRI, and in utero electroporation lineage tracing.

#### 8.1.4. Behavioral Studies in Offspring

If high-PUFA cooking oil exposure alters brain development, animals should exhibit measurable neurobehavioral phenotypes attributable to neurodevelopmental pathologies. Again, using the autism spectrum as an example, the following exposure outcomes could be assessed:

- Social-interaction deficits;
- Sensory hypersensitivity or gating abnormalities;
- Repetitive behaviors;
- Impaired vocalization development.

Such outcomes would parallel established environmental autism models (e.g., those of valproate, ethanol and maternal immune activation).

### 8.2. Human Studies

Parallel human research is required to translate mechanistic and animal findings into actionable prenatal guidance:

#### 8.2.1. Improved Dietary-Exposure Assessment Tools

Unfortunately, current food-frequency questionnaires collapse all plant oils into broad categories and often conflate PUFA from added oils with PUFA from whole foods. Therefore, new or already established instruments should capture:

- The type of oil used for home and commercial cooking practices;
- Frequency of consuming foods prepared in repeatedly heated oils (e.g., fries, tortilla chips, crackers, pastries, fast-food entrées, etc.);
- Degree of oil reuse or heating intensity;
- Use of oxidation-resistant fats and MUFAs (butter, ghee, tallow, high-oleic acid content oils, etc.).

Without these variables, epidemiologic studies cannot detect associations uniquely attributable to aldehydic load ingestion.

#### 8.2.2. Maternal Biomarker Development

Biomarkers that may reflect maternal oxidative stress and aldehyde exposure include:

- A range of circulating aldehydes and their adducts, e.g., mercapturate conjugates from the metabolism of α,β-unsaturated aldehydes;
- Oxylipin and oxLAM signatures;
- Glutathione depletion or redox shifts;
- The placental aldehyde-adduct burden.

Establishing reliable biomarkers is essential for linking actual exposure to fetal outcomes.

#### 8.2.3. Pregnancy Cohort Studies Incorporating LOP and Aldehyde-Specific Exposure Measures

Large observational cohorts should examine whether higher aldehyde intake—as estimated by improved dietary tools or biomarker profiles—is associated with:

- Altered fetal growth trajectories;
- Adverse obstetric outcomes (e.g., pre-eclampsia, gestational diabetes);
- Neonatal neurobehavioral measures;
- Early childhood developmental milestones and sensory-processing profiles.

Such studies would determine whether aldehydes function as population-level prenatal risk factors.

#### 8.2.4. Mechanistic Imaging and Placental Studies

Advanced imaging and histological analysis could evaluate whether maternal aldehyde exposure correlates with:

- Placental vascular changes;
- Altered nutrient transport;
- Inflammatory markers;
- Neonatal brain microstructural measures (e.g., diffusion MRI).

These relationships could reveal early biomarkers of aldehyde-associated neurodevelopmental disruption.

## 9. Conclusions: Implications for Maternal–Child Health and Nutrition Policy

PUFA-rich cooking oils in particular produce significant quantities of harmful LOPs when fried at high temperatures, and PUFAs are, of course, more susceptible to lipid peroxidation than MUFAs. Conversely, saturated fatty acids (SFAs) are almost completely resistant to lipid peroxidation, and thus oils or frying media containing high levels of it offer the most preferred oils for frying practices. Similarly, MUFA-rich oils with high or very high levels of MUFAs are recommended since they produce much lower LOP levels during frying when compared to PUFA-rich frying media (e.g., sunflower oil at high temperature, Figure 3).

The role of toxic aldehydic LOPs in the development and prevalence of non-communicable diseases (NCDs) is now very well documented by a large body of evidence [15,16]. We also accept that the excessive consumption of SFAs may present some hazards to human health, and in the current paper, we wish to provide an overall health-balanced approach.

Neurodevelopmental disorders are increasing in prevalence and impose substantial burdens on individuals, families, and society, including lifelong healthcare costs, reduced productivity, and significant caregiver strain. Despite extensive research efforts, many proposed environmental risk factors lack clear biological mechanisms or are not readily modifiable, limiting their translational impact.

We have shown that LOPs, particularly certain well-studied aldehydes, can disrupt brain cytoskeletal dynamics, and their consumption during fetal life may therefore have devastating effects that have not been widely appreciated in the clinical nutrition literature. Compounding this problem, attention has been concentrated on acrolein and the 4-hydroxy-(*E*)-2-alkenal HNE, despite the fact that the latter oxygenated and unsaturated aldehyde is only generated in cooking oils at very low levels overall (Figure 3). Meanwhile, the (*E*,*E*)-alka-2,4-dienals represent a class of aldehydes with a more reactive chemical structure and an oral consumption TTC value, which is estimated to be exceeded 5-fold or so by this compound on a daily basis. Our data shows that this aldehydic LOP can account for as much as 20–35 mol % of the total aldehyde content in fried cooking oils (that present in French fries fried in a PUFA-rich cooking oil is approximately 35 mol.%). Until now, this class of α,β-unsaturated aldehydes has received comparatively little or no attention in developmental toxicology.

Closing the disciplinary gap that has to date obscured dietary aldehyde exposure will require coordinated integration across nutritional epidemiology, lipid oxidation chemistry, toxicology, and developmental neurobiology. This includes improving dietary exposure assessments to distinguish between cooking oil types and preparation methods, incorporating measurements of LOP- and aldehyde–adduct biomarkers into human studies, and designing experimental models that connect electrophilic reactivity with neurodevelopmental end-points. In principle, for large population studies, such biomarkers could be monitored in pioneering wastewater sample analysis studies, in addition to those featuring common, patient-specific biofluids. Indeed, a very recent investigation performed by Dong et al. [153] revealed dietary marker profiles for wealth, immigration and coastal proximity. However, it is very important to note that measuring the degree of peroxidation in either food systems or human consumers using only one or two markers may indeed represent an erroneous strategy, considering volatile determination techniques that assist in the provision of information regarding at least several markers, which provide us with much more valuable information.

Shared methodological standards and cross-disciplinary funding priorities will be essential to trace the full pathway from dietary intake to molecular mechanisms to developmental outcomes. Establishing such an integrated framework—conceptualized here as ***Dietary LOP Pathology***—would enable systematic evaluation of diet-derived electrophiles as prenatal exposures.

Reducing aldehyde exposure may also confer valuable benefits beyond neurodevelopment. These compounds promote oxidative stress, mitochondrial dysfunction, endothelial injury, and systemic inflammation—pathways implicated in gestational diabetes, pre-eclampsia, and other metabolic complications of pregnancy. Improving the aldehyde profile of the maternal diet could therefore yield parallel benefits for maternal health and fetal outcomes.

Given the widespread use of refined culinary oils in modern diets, and the feasibility of reducing aldehyde exposure through changes in food preparation and ingredient selection, this will represent a practical and underexplored opportunity for maternal–child health research and policy.

## Figures and Tables

**Table 1:** *cis*-/*trans*- and corresponding (*Z*)-/(*E*)- nomenclature for unsaturated aldehydic LOPs, with typical examples. Also listed are the Threshold of Toxicological Concern (TTC) values [59] in (µg/kg bw) per day and (µg/70 kg bw) per day for the examples given. The percentage TTC values of the aldehyde content in a 1 oz (28.35 g) serving of potato chips are also provided. To date, TTC values for pregnant women have not been separately validated through recommended experimental protocols. Abbreviations: LMM, low-molecular-mass.

Aldehyde Classification (*cis*/*trans*-isomers)	Standard (*E*)/(*Z*) IUPAC Nomenclature System for Unsaturated Aldehydes	Typical Example	TTC (µg/kg bw) per Day	TTC (µg/70 kg bw) per Day	Amount in 1 oz (28.35 g) **	% of (µg/70 kg bw) per Day TTC (1.00 oz Serving) **
**Acrolein (lowest homolog Alkenal)**	Acrolein	Acrolein	0.0025 (Genotoxic/Carcinogenic Category)	0.175 (Genotoxic/Carcinogenic Category)	0.65 µg [a]	373% [a]
*trans*-2-Alkenals *	(*E*)-2-Alkenals *	(*E*)-2-Octenal *	9 (Class II) *	630	467.6 µg [b]	74%
***trans*,*trans*-Alka-2,4-Dienals**	(*E*,*E*)-Alka-2,4-Dienals	(*E*,*E*)-Deca-2,4-Dienal	1.5 (Class III)	105	537.5 µg [c]	512%
**4,5-Epoxy-*trans*-2-Alkenals**	4,5-Epoxy-(*E*)-2-Alkenals	4,5-Epoxy-(*E*)-2-decenal	0.0025 (Genotoxic/Carcinogenic Category)	0.175 (Genotoxic/Carcinogenic Category)	No data	No data
**4-Hydroxy-*trans*-2-Alkenals**	4-Hydroxy-(*E*)-2-Alkenals	4-Hydroxy-(E)-2-nonenal (HNE)	1.5 (Class III)	105	~2.2–9.4 µg [d]	~2–9%
**4-Hydroperoxy-*trans*-2-Alkenals**	4-Hydroperoxy-(*E*)-2-Alkenals	4-Hydroperoxy-(*E*)-2-nonenal (HPNE)	0.0025 (Genotoxic/Carcinogenic Category)	0.175 (Genotoxic/Carcinogenic Category)	No data	No data
***cis*,*trans*-Alka-2,4-Dienals**	(*Z*,*E*)-Alka-2,4-Dienals	(*Z*,*E*)-Deca-2,4-dienal	1.5 (Class III)	105	73.0 µg	69%
***n*-Alkanals (Standard MM)**	na	*n*-Hexanal	30 (Class I)	2100	476.8 µg [b]	23%
**LMM *n*-Alkanals**	na	Propanal/*n*-Butanal	30 (Class I)	2100	No data	No data
*cis*-2-Alkenals *	(*Z*)-2-Alkenals *	(E)-2-Nonenal *	23.3 (Class I) or 9 (Class II) *	1400 (Class I) or 630 (Class II) *	No data	No data
**Acetaldehyde**	na	Acetaldehyde	0.0025 (Genotoxic/Carcinogenic Category)	0.175 (Genotoxic/Carcinogenic Category)	No data	No data
**Formaldehyde**	na	Formaldehyde	Not available, since robust carcinogenicity data are already accessible.	na	No data	No data
**Malondialdehyde (MDA)**	na	MDA	30 (Class I)	2100	~ 0–12.3 µg [e]	~ 0–0.58%

**Table 1 Notes:** *trans*-2-Alkenals ((*E*)-2-alkenals) * and *cis*-2-Alkenals ((*Z*)-2-alkenals) * were classified as Class II in view of a reactive electrophilic Michael addition attack site at position C3, in addition to the position C1 aldehydic-CHO function. ** 1.00 oz serving per day is the average amount consumed by a UK adult averaged throughout the year (ca. 30 g). Typical large-size servings are 5 oz (~150 g). [a] Scaled from a directly measured value in the literature, not from oil concentration [60], as cited in Jiang et al. [61]. Acrolein was measured in potato chips fried in rapeseed oil; this level was 23 µg/kg. Scaled to a 1 oz (0.02835 kg) serving: 0.02835 kg × 23 µg/kg = 0.65 µg. Caveat: this measurement is for potato chips (crisps), not French fries, so it is the best available proxy rather than a fries-specific figure. [b] Stated directly in Section 2.6 for a 154 g serving, but scaled here to 1 oz (28.35 g) using the ratio 28.35/154 (=0.1841). [c] Stated for (*E*,*E*)-deca-2,4-dienal, and scaled as in [b]. [d] Derived, not stated by the authors: Table 2 gives 0.5–2.1 µmol/kg in French fries (Csallany et al. (Ref. [62]); converted using the HNE molecular weight of 156.22 g/mol, and scaled to a 28.35 g (1 oz) serving. [e] Derived, not stated by the authors: Table 2 gives 0–6 µmol/kg in French fries; converted using MDA molecular weight of 72.06 g/mol, and scaled to a 28.35 g (1 oz) serving.

**Table 3:** Documented protein targets of the three best-characterized LOP-derived aldehydes, illustrating the breadth of reactivity despite the limited number of aldehydes studied to date.

Targets	Malondialdehyde (MDA)	4-Hydroxy-(*E*)-2-Nonenal (HNE)	Acrolein	Total (Unique)
**Documented protein targets**	32	~77	28	~95–100 ^a^
**Targets confirmed in brain tissue/cells**	17	24	Not studied ^b^	~32–33
**Cytoskeletal element targets**	6 (β-actin, γ-actin, α-tubulin, β-tubulin 2, vimentin, GFAP)	4 (β-actin, α-tubulin, β-tubulin, vimentin)	2 (β-actin, keratin type 1)	7 ^c^

^a^ Data provided accounts for targets shared across aldehydes and between sources. ^b^ No brain-tissue or brain-derived-cell adduction studies for acrolein were identified in the sources reviewed here; this reflects a gap in the literature rather than a demonstrated absence of reactivity, particularly when considering acrolein’s established reactivity profile and documented adduction of homologous or functionally analogous targets by MDA and HNE. ^c^ Defined as core structural cytoskeletal polymer components (actin isoforms, tubulin isoforms, and intermediate filaments). In addition to those listed, collapsin regulatory protein 2 regulates cytoskeletal dynamics, particularly microtubule assembly and axon growth, and its disruption by aldehydes would also be potentially detrimental to neurodevelopmental processes dependent on cytoskeletal function. Sources: Refs. [122,123].

## Data Availability

No new data were created or analyzed in this study. Data sharing is not applicable to this article.

## Supplementary Materials

The following supporting information can be downloaded at: https://www.mdpi.com/article/10.3390/molecules31193530/s1. Figure S1: Expanded aldehydic-CHO proton (9.46–9.79 ppm) region of 600 MHz 1H NMR spectra of a typical canola oil product exposed to thermal stressing episodes according to our laboratory simulated shallow frying episodes (LSSFEs); samples were collected for ^1^H NMR analysis at the 0 (unheated), 5, 10, 20, 30, 60 and 90 min. time-points.; Figure S2: Expanded 9.46-9.79 ppm (aldehydic proton) region of the 600 MHz ^1^H NMR spectrum of a deuterochloroform (C_2_HCl_3_) extract of UK-available packeted potato crisp snacks, showing the ready detection of four classes of aldehydes in this product. Table S1: Boiling-points (B.pts) of some typical aldehydic LOPs (at 760 mmHg) liberated from peroxidised UFAs, particularly PUFAs, which are valuable for estimating the relative quantities of volatile aldehydes in the vapour phase during frying episodes.; Table S2: Assignments of ^1^H NMR -CHO signals of aldehydes (secondary LOPs) present in the ^1^H NMR profiles of culinary oils, including their chemical shift values (δ in ppm), and multiplicities.
