# Supplementary material for: Cooking Oil-Derived Aldehydes: Overlooked Components of the Western Diet with the Potential to Disrupt Fetal Brain Development
Source: Molecules. 2026 Oct 3;31(19):3530. doi: 10.3390/molecules31193530 (PMC13666609; doi:10.3390/molecules31193530)
Supplement: Supplementary file 1 [file molecules-31-03530-s001.zip › molecules-4481080-supplementary.pdf]

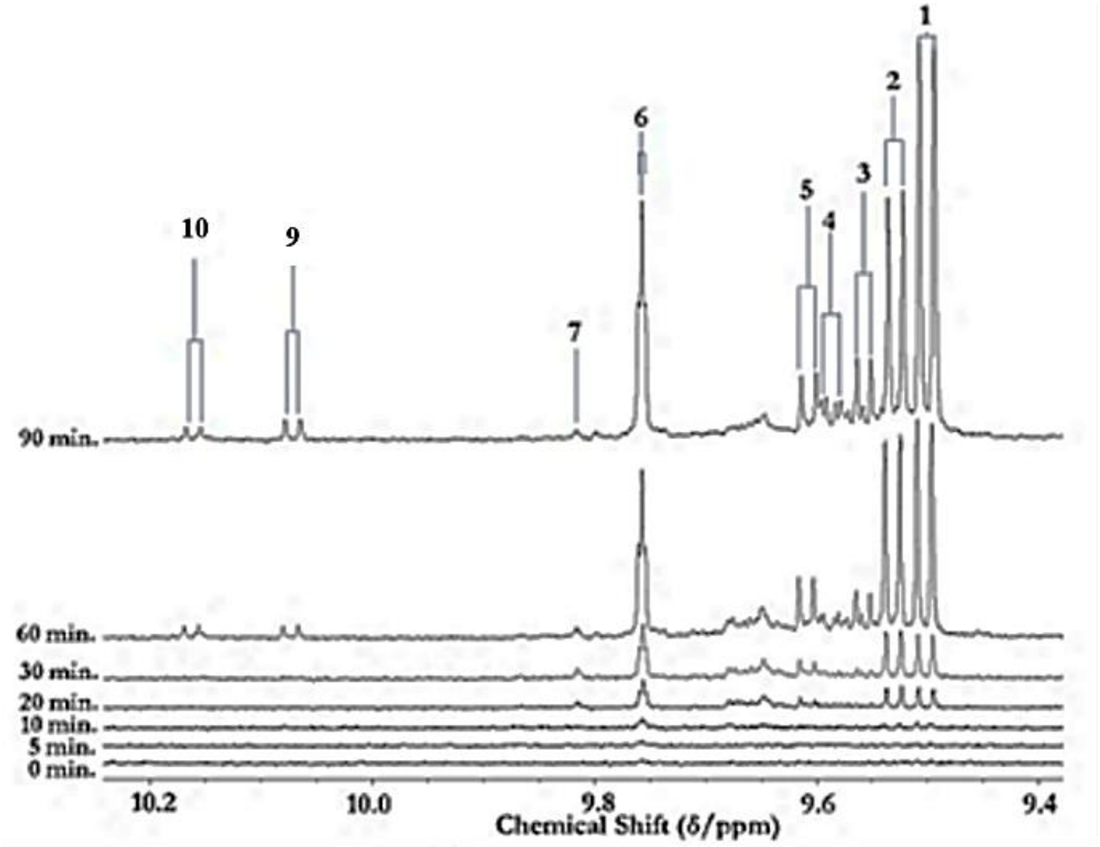

Figure S1. Expanded aldehydic-CHO proton (9.46–9.79 ppm) region of 600 MHz ^{1}H NMR spectra of a typical canola oil product exposed to thermal stressing episodes according to our laboratory-simulated shallow frying episodes (LSSFEs); samples were collected for ^{1}H NMR analysis at the 0 (unheated), 5, 10, 20, 30, 60 and 90 min. time-points. All resonances visible are doublets (J = 7.63 Hz for *trans*-2-alkenals), with the exception of the *n*-alkanal one which is a triplet (J = 1.82 Hz). Abbreviations: 1, (*E*)-2-alkenals (*doublet*); 2, alka-(*E,E*)-2,4-dienals (*doublet*); 3, 4,5-Epoxy-(*E*)-2-alkenals (*doublet*); 4, 4-hydroxy-/4-hydroperoxy-(*E*)-2-alkenals (overlapping *doublets*); 5, alka-(*Z,E*)-2,4-dienals (*doublet*); 6, higher homologue *n*-alkanals such as *n*-hexanal (*triplet*); 7, 4-oxo-*n*-alkanals (*triplet*, J = 1.78 Hz); 9, (*Z*)-2-alkenals (*doublet*); 10, unidentified aldehyde, presumably a substituted (*Z*)-2-alkenal derivative. The total SFA, MUFA and PUFA contents (w/w) of the unheated (control) canola oil was 7.0, 64.4 and 28.5% (w/w) respectively, that of PUFAs representing 20.7% for linoleate, and 7.8% for linolenate. This diagram was originally published in Ref. [53], and is reproduced here in full.

Immediately following collection, the lipid-soluble antioxidant 2,5-di-*tert*-butylhydroquinone (DTBHQ) was added to each oil sample at a final added concentration of 10.00 mmol/kg in order to block or retard the further generation of aldehydes and their HPM and CHPD precursors during periods of storage, and sample preparation at ambient temperature. Samples were prepared for ^{1}H NMR analysis within 2 hr. after collection, and were stored in sealed containers within a light-excluded zone at 4°C whilst awaiting analysis.

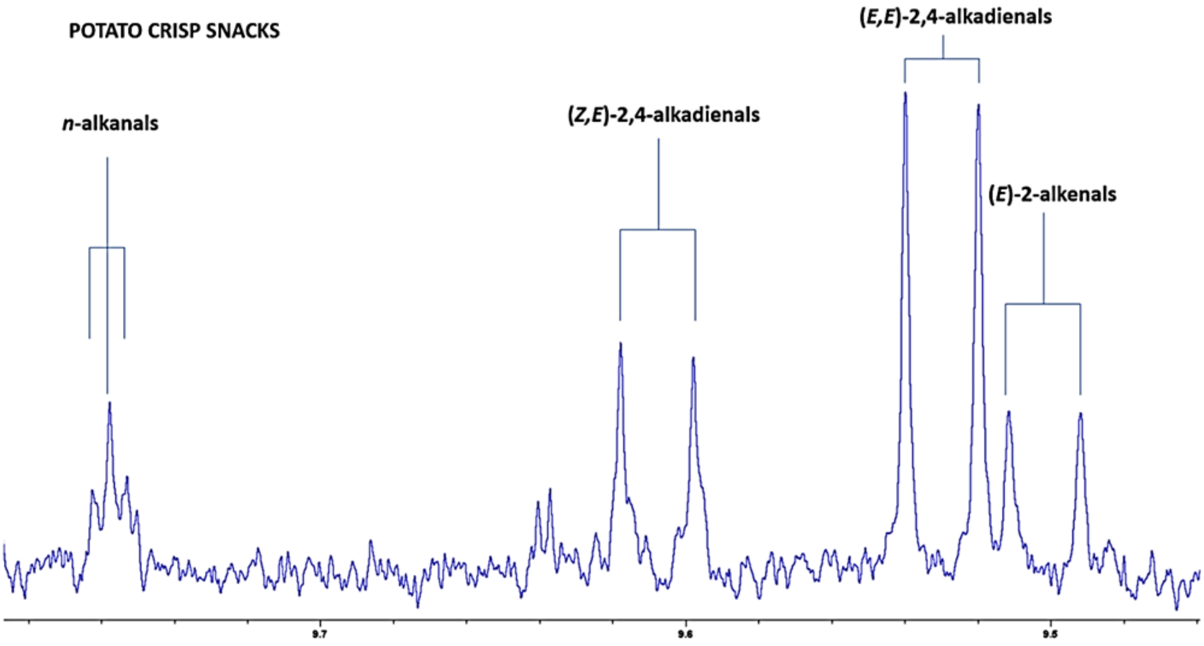

Figure S2. Expanded 9.46-9.79 ppm (aldehydic proton) region of the 600 MHz ^{1}H NMR spectrum of a deuterochloroform (C_2HCl_3) extract of UK-available packeted potato crisp snacks, showing the ready detection of four classes of aldehydes in this product. Measured weights (*ca.* 100 mg) of this food product were extracted with 800 µL volumes of deuterochloroform (C^2HCl_3) containing a known concentration of 1,3,5-tribromobenzene (δ = 7.54 ppm) as an internal chemical shift and quantitative ^{1}H NMR standard.

TABLE S1. Boiling-points (B.pts) of some typical aldehydic LOPs (at 760 mmHg) liberated from peroxidised UFAs, particularly PUFAs, which are valuable for estimating the relative quantities of volatile aldehydes in the vapour phase during frying episodes. Their triacylglycerol (TAG) sources are also listed [28]. Abbreviations: OA, oleic acid acylglycerol chains; MDA, malondialdehyde; (*E*),trans-configuration. *2-heptenal isomers are derived from alka-2,4-dienal decomposition, along with acetaldehyde, hexanal, acrolein, butenal, 2-heptenal, 2-octenal, benzaldehyde, glyoxal,
and (*E*)-2-buten-1,4-dial [66].

Aldehyde	B.pt (◦C)	Acylglycerol chain or alternative TAG sources
**Formaldehyde**	-19	Decomposition of MDA
**Acetaldehyde**	20	Alka-2,4-dienal decomposition
**Propanal**	49	ALA
***n*-Pentanal**	102-103	LA
***n*-Hexanal**	131	LA/Alka-2,4-dienaldecomposition
***n*-Octanal**	173	LA/OA
***n*-Nonanal**	79-81	OA
***n*-Decanal**	213	OA
**Acrolein**	53	ALA/Glycerol Backbone/Alka-2,4-dienal decomposition
**(*E*)-2-Heptenal***	166	Alka-2,4-dienal decomposition
**(*E*)-2-Octenal**	84-86	LA/Alka-2,4-dienal decomposition
**(*E*)-2-Nonenal**	205	LA
**(*E*)-2-Decenal**	230	OA
**(*E*)-2-Undecenal**	234	OA
**(*E,E*)-Hepta-2,4-Dienal**	177	ALA
**(*E,E*)-Deca-2,4-Dienal**	115	LA
**4-Hydroxy-(*E*)-2-hexenal**	233.5±23	ALA
**4-Hydroxy-(*E*)-2-nonenal**	276±23	LA
**Malondialdehyde**	108±23	ALA

Table S2. Assignments of ^{1}H NMR -C<u>H</u>O signals of aldehydes (secondary LOPs) present in the ^{1}H NMR profiles of culinary oils, including their chemical shift values (δ in ppm), and multiplicities. Abbreviations: *d*, doublet; *t*, triplet.

Chemical Shift Value (ppm)	Multiplicity	Aldehydic LOP Assignment
9.48–9.51	*d*	(*E*)-2-Alkenals
9.51–9.54	*d*	(*E*,*E*)-2,4-Alkadienals
9.54–9.57	*d*	4,5-Epoxy-(*E*)-alkenals
9.57–9.59	*d*	4-Hydroxy-(*E*)-2-alkenals
9.58–9.59	*d*	4-Hydroperoxy-(*E*)-2-alkenals
9.59–9.62	*d*	(*Z*,*E*)-2,4-Alkadienals
9.742–9.77	*t*	*n*-Alkanals
9.78–9.80	*t*	4-Oxo-*n*-alkanals
9.80–9.82	*t*	*n*-Alkanals of low molecular mass (LMM) (e.g., ethanal, propanal and butanal)
10.05–10.08	*d*	(*Z*)-2-Alkenals
10.14–10.17	*d*	Unassigned Alkenal species
